# TERT promoter alterations could provide a solution for Peto’s paradox in rodents

**DOI:** 10.1038/s41598-020-77648-0

**Published:** 2020-11-30

**Authors:** Balázs Vedelek, Asha Kiran Maddali, Nurgul Davenova, Viktor Vedelek, Imre M. Boros

**Affiliations:** 1 Department of Biochemistry and Molecular Biology, University of Szeged, Szeged, Hungary; 2 Institute of Biochemistry, Biological Research Centre, Szeged, Hungary; 3 Institute of Genetics, Biological Research Centre, Szeged, Hungary; 4 Department of Genetics, University of Szeged, Szeged, Hungary

**Keywords:** Gene regulation, Cancer prevention, Senescence, Telomeres

## Abstract

Cancer is a genetic disease caused by changes in gene expression resulting from somatic mutations and epigenetic changes. Although the probability of mutations is proportional with cell number and replication cycles, large bodied species do not develop cancer more frequently than smaller ones. This notion is known as Peto’s paradox, and assumes stronger tumor suppression in larger animals. One of the possible tumor suppressor mechanisms involved could be replicative senescence caused by telomere shortening in the absence of telomerase activity. We analysed telomerase promoter activity and transcription factor binding in mammals to identify the key element of telomerase gene inactivation. We found that the GABPA transcription factor plays a key role in TERT regulation in somatic cells of small rodents, but its binding site is absent in larger beavers. Protein binding and reporter gene assays verify different use of this site in different species. The presence or absence of the GABPA TF site in TERT promoters of rodents correlates with TERT promoter activity; thus it could determine whether replicative senescence plays a tumor suppressor role in these species, which could be in direct relation with body mass. The GABPA TF binding sites that contribute to TERT activity in somatic cells of rodents are analogous to those mutated in human tumors, which activate telomerase by a non-ALT mechanism.

## Introduction

It has been observed that between different species, animal body size and cancer incidence do not correlate with each other, despite the fact that larger biomasses are assembled from more cells and would, therefore, be expected to incur an increased probability of mutations^1^. Within species, however, the incidence of cancer is higher in larger individuals^2^. To explain this contradiction it was suggested that animal species with larger bodies have developed more strict tumor suppressor mechanisms than those with smaller ones^3,4^. One of these mechanisms is the regulation of telomere length through telomerase reverse transcriptase (TERT) enzyme activity^5–9^. In human stem cells the telomerase is active, however in somatic cells the catalytic subunit of the enzyme is transcriptionally silenced^10^. In the absence of functional telomerase, chromosome ends shorten with each cell cycle as a result of the end replication problem^11,12^. Depleted telomeres induce apoptosis or cause cell cycle arrest, resulting in a cell state referred to as replicative senescence. The tumor suppressor role of replicative senescence is still debated, however it is supported by numerous wet lab experiments^8,13–16^ (see^9^ for a review). A recent report on whole genome sequence analysis of a very large number of tumor samples also strengthens the tumor suppressor role of senescence induced by telomere attrition^17^. The reason for the debate concerning the tumor suppressor role of replicative senescence is its similarity to induced senescence, which occurs from causes other than telomere shortening in cellular homeostasis during ageing of cells. With certain limitations induced senescence can be considered a tumor suppressor mechanism^18^; however, it is a “double-edged sword” since senescent cells could both improve immune response and promote tumor progression—for reviews on this see^19,20^.

In somatic cells of mice and other small rodents the TERT promoter is active^21^. In these rodents, the tumor suppressor effect of telomere shortening may never be essential or take place because their lifespan is short and fewer number of cell divisions are necessary to reach the final body mass compared to large mammals^6,7^. In general, in mammalian species the length of telomeres in somatic cells is in correlation with telomerase activity and inversely correlates with body mass, but not with lifespan^6,7,22^. Nevertheless diverse tumor suppressor mechanisms act in small but long lived animals, like naked mole rats or squirrels^23^.

Replicative senescence resulting from telomere shortening is the consequence of telomerase inactivation in somatic cells. Early experiments with TERT null mutant mice resulted in contradicting data regarding the tumor suppressor effect of telomere shortening. In the absence of TERT expression telomeres indeed shortened in mice, but the overall tumor incidence increased^24^. However, telomere depleted mice were resistant to skin cancer^25^. The increased cancer incidence in TERT KO mice could be explained by telomere loss in stem cells compromising the immune system, thereby allowing more cancer cells to avoid immune surveillance. Several other severe phenotypes characterize TERT KO mice, indicating the importance of telomere maintenance in stem cells^26^. A recent report offers further insight to this puzzle by pointing out the importance of transient upregulation of TERT for appropriate senescence response^27^. It was found that cells from animals in which the telomere length was shortened due to down-regulation of TERT reached senescence earlier than cells from their wild type relatives; however, a transient TERT up-regulation was necessary for the proper senescence response. Without TERT, cells were more frequently immortalized using the alternative telomere elongation (ALT) mechanism, which caused increased tumor incidence. In contrast, when TERT gene was present, fewer cells were immortalized due to the proper senescence response. Cells only occasionally escaped from senescence, which was associated with telomerase activation. Only 10% of human tumors maintain telomeres by ALT and 90% use reactivation of TERT, which reveals the importance of replicative senescence in tumor suppression^28–31^.

During the evolution of rodents the inactivation and activation of telomerase in somatic cells may have happened multiple times. Telomerase in platypus is found to be active^32^. It is believed that it became inactivated in early placental mammals but reactivated in a common ancestor of rodents, and it is active in most recent rodent species^6,22^. Exceptions are beavers and capybaras, in which the telomerase activity is lower than in other rodents. These species have larger body masses, at which replicative senescence could be beneficial. We hypothesized that telomerase inactivation in beavers and capybaras originates from changes in the promoter of the TERT gene. Revealing the importance of a cis-acting element in TERT gene inactivation highlights not only an evolutionary mechanism that allowed longevity and therefore increased fitness of large bodied animals, but also our vulnerability to acquired mutations providing an ideal cellular environment for the development of cancer.

Due to the rapid development of sequencing methods, genome sequence data are available from a wide variety of mammals. By using these and data on telomerase activity, it is possible to identify potential transcription factor binding sites that could explain the activation or inactivation of TERT expression in different mammals. By identifying these transcription factors, a mechanism can be proposed to explain the evolution of replicative senescence as a tumor suppressor mechanism in larger animals.

In this study we performed comparative TERT promoter analysis of different mammals with known TERT activity in somatic cells. We found that GABPA is a key factor in determining TERT transcription status in mammals of different evolutionary groups, similarly to certain human cancer cells. Our results support the hypothesis that a mutation in the TERT promoter is sufficient for the inactivation of the gene in somatic cells, triggering replicative senescence as a tumor suppressor mechanism. Therefore, the presence or absence of the GABPA binding site in the TERT promoter depicts a mechanism that might solve Peto’s paradox.

## Results

### GABPA, Elk1, E47 and GATA3 binding sites represent differences between active and inactive TERT promoters

To study the promoter regions of the TERT gene we extracted 1000 bp long sequences upstream from the TERT start codon of 28 placental mammalian species from public databases. In 14 of these species telomerase is active, while in the rest telomerase is inactive in somatic cells. Data on telomerase activity were collected from the literature, and reflect mainly TERT activity determined in fibroblasts from skin, kidney, lung or cornea^6,7,22,32–34^. An alignment of the sequences reveals that the TERT promoter sequence is rapidly evolving, therefore a comparison among them on nucleic acid level is challenging (Supplementary Fig S1). However, transcription factor (TF) binding sites are more conserved. To identify and compare TF sites within these promoter regions we analysed the sequences by Match and TFbind software^35^. The identified TF binding sites were scored based on their similarity to the consensus site matrices and filtered based on their score. Strong binding motifs that ranked above cut-off values were considered in further analysis. The frequency of predicted TF binding sites was summarised and analysed by random-sampling bootstrap method (Supplementary Table S1). In cases where binding sites showed near binary distribution we also performed Fisher’s exact tests (Supplementary Table S2). The transcription factor matrices, which were identified by at least two prediction methods or statistical probes, are summarised in Table 1.

**Table 1:** Summary of transcription factor matrices that were found to be significant by at least two approaches.

Factor (Matrices ID)	Prediction method	Statistics	H_0_: inactive > active (p-value)^a^	H_0_: inactive = active (p-value)^a^	H_0_: inactive < active (p-value)^a^
GABPA (V$NRF2_01)	Match	Bootstrap	**0.0000**	0.0834	0.9172
	Match	Fisher	–	**0.0002**	–
	TFBind	Bootstrap	**0.0000**	**0.0272**	0.9710
	TFBind	Fisher	–	**0.0213**	–
E47 (V$E47_01)	Match	Bootstrap	0.6024	0.3866	**0.0008**
	TFBind	Bootstrap	0.9136	0.0886	**0.0000**
	TFBind	Fisher	–	**0.0183**	–
GATA3 (V$GATA3_01)	TFBind	Bootstrap	**0.0008**	0.2022	0.7892
	TFBind	Fisher	–	**0.0461**	–
Elk1 (V$ELK1_02)	Match	Bootstrap	**0.0026**	0.2010	0.8074
	TFBind	Fisher	–	**0.0407**	–

^**a**^Probabilities for three zero hypotheses were calculated. Low values (< 0.05, bold) indicate hypothesis deemed to be failed.

**Figure 1:**
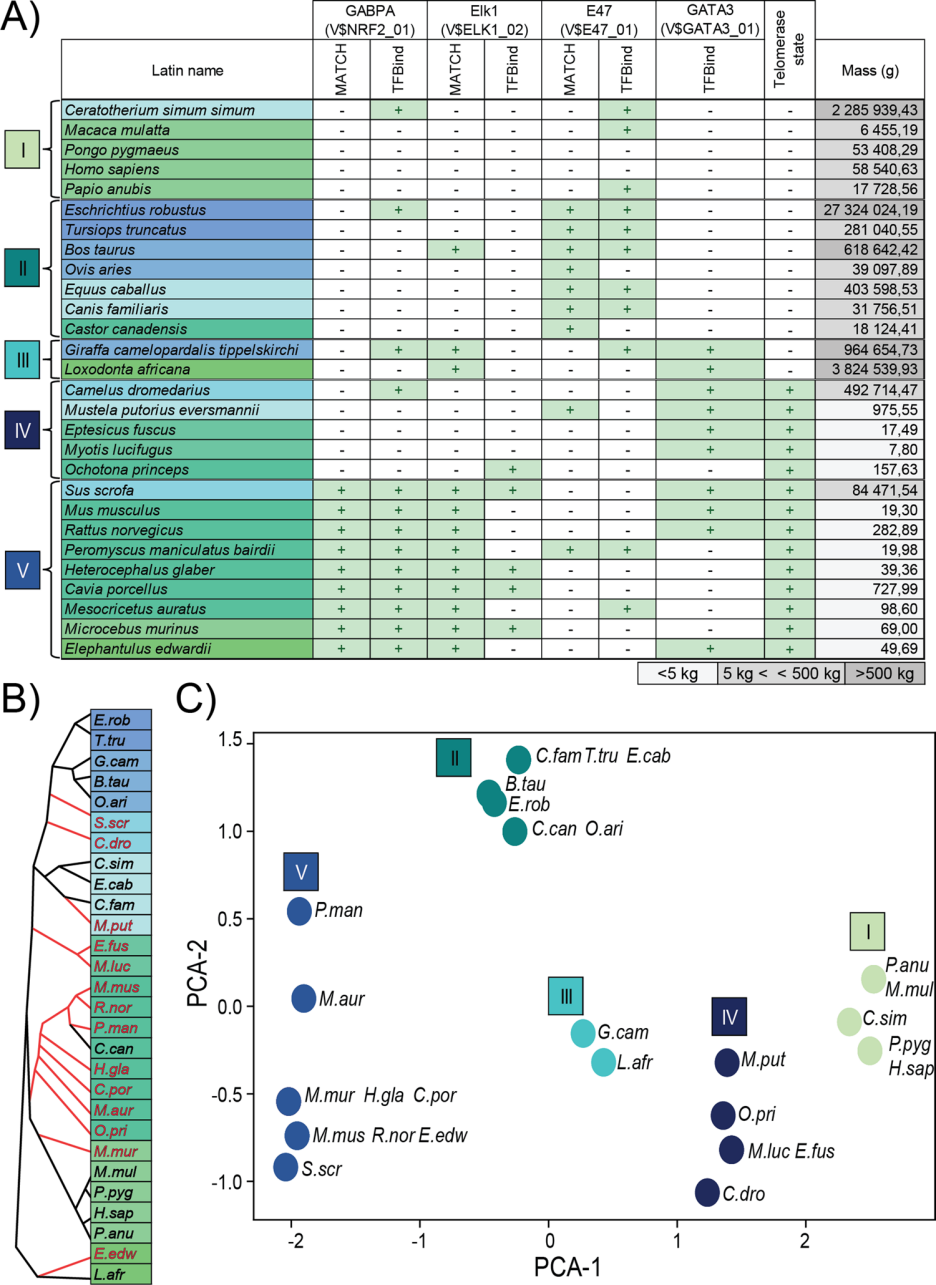
Telomerase state and transcription factor binding site distribution among 28 studied mammalian species. (**A**) The 28 species were clustered into 5 groups by k-means clustering based on predicted transcription factor binding sites and telomerase state (active+/inactive−). The background coloring of species names represents phylogenetic relations. (**B**) The phylogenetic relationship of the 28 species involved in the study. Red indicates species with active, black with inactive telomerase in somatic cells. (**C**) Principal component analysis (PCA) shows the clear separation of the clusters. Coloring corresponds to groups which were determined by the clustering.

By the analysis described above, we identified four factors that could have a role in the different regulation of the TERT gene in small and large-bodied mammals. The GABPA (or NRF2) and ELK1 proteins are Ets transcription factor family members. E47 (or TCF3) is a member of the E protein family of helix-loop-helix transcription factors, and GATA3 is a member of the GATA transcription factor family. The E47 (V$E47_01) binding site is more likely to be present in inactive TERT promoters, while the other three sites (V$NRF2_01, V$Elk1_02, V$GATA3_01) are mostly found on active promoters. We performed k-means clustering multiple times on the data using the occurrence of transcription factors and the telomere state as a base. The clustering which best represents phylogenetic relations is presented in Fig. 1. (Other possible clusterings are listed in Supplementary Table S3). In groups I and II the telomerase is inactive; NRF2 and ELK1 sites are absent, but E47 binding sites are present. In group III the telomerase is inactive despite the presence of ELK1 and GATA3 sites. Group IV is characterized by active telomerase and the presence of GATA3 transcription factor sites, but no other studied site is present in these promoters. Telomerase is also active in group V where GABPA and ELK1 binding sites are present, however E47 sites are rare.

**Figure 2:**
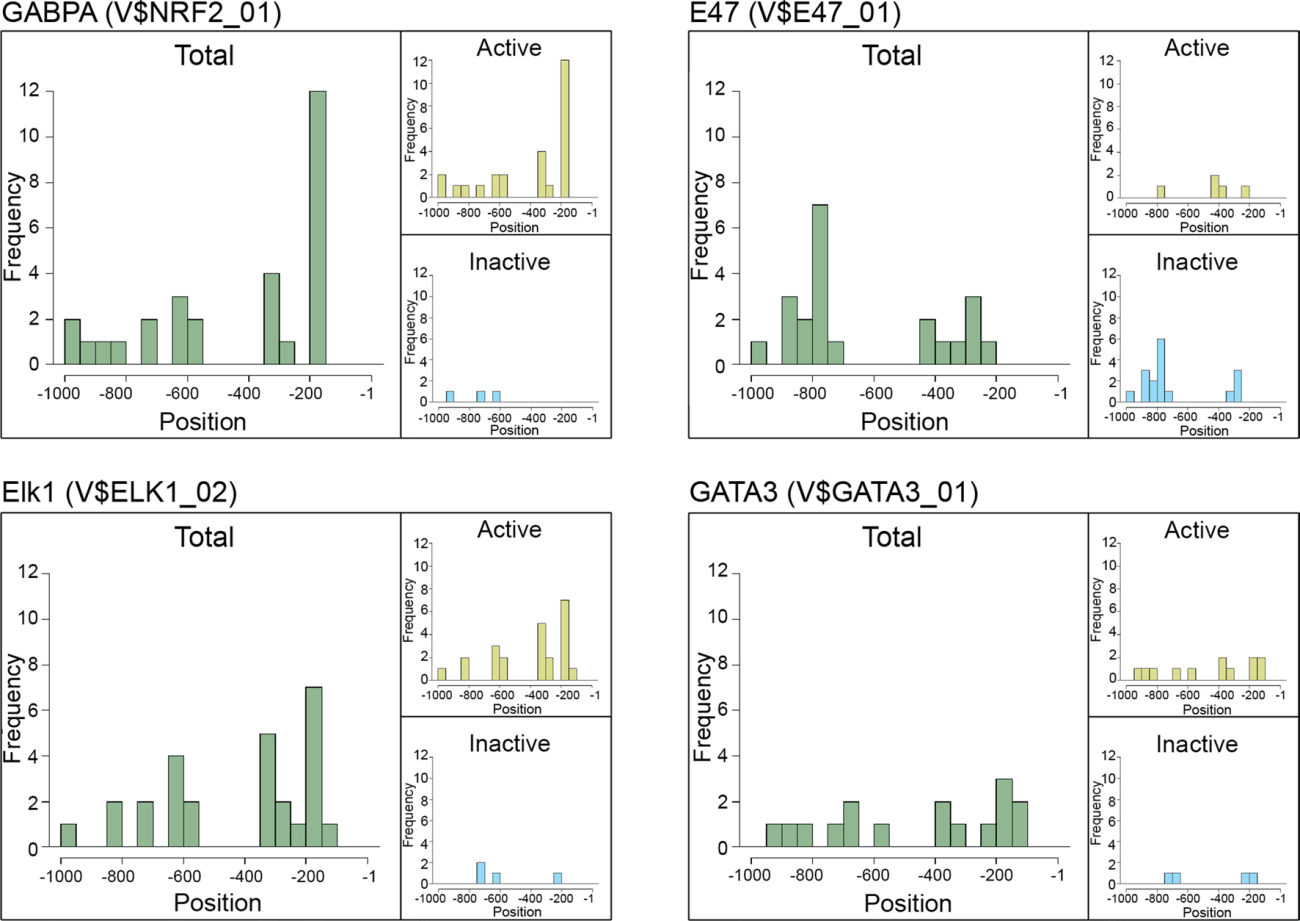
Distribution of transcription factor binding sites identified by in silico analysis within the − 1000 to − 1 region upstream of the translation start in TERT promoters of 28 mammalian species. Green bars: distribution of the binding sites of the factor indicated on top of the plot at the 28 TERT promoter region. Yellow bars: distribution of the sites of the indicated factor on active promoters; blue bars: distribution of binding sites on inactive promoters.

Next, we examined the positional distribution of these transcription factor binding sites on the promoter sequences of different species (Fig. 2). GABPA sites are located frequently between the − 150 and − 200 bp region upstream of the START codon. ELK1 sites also seem to be present in this region (ELK1 is also an Ets factor with highly similar recognition sequence) but in a more scattered pattern. E47 sites are more often present in the inactive promoters, about 800 bp upstream of the START codon. GATA3 sites show an even distribution, and like the GABPA and Elk1 sites they can be found in active promoters.

Based on our statistics on binding site presence and binding site distribution, we concluded that the GABPA TF could be the distinguishing factor between TERT active and inactive states. Our conclusion is supported by the notion that in human tumor cells a single mutation either at 146 or at 124 nucleotides upstream of the START codon of TERT is enough to reactivate telomerase^29,36^. C to T mutation(s) within either or both of these sites creates a binding site for the GABPA transcription factor. Binding of the GABP complex of GABPA and GABPB results in the spreading of euchromatic chromatin structure to the promoter region and consequent transcriptional activation^29,37^.

We also tested the conservation of the GABPA protein among mammals to see the conservation of its consensus binding site. We found that the protein is highly conserved, with an identical DNA binding domain in most cases (Supplementary Fig S2). Only mouse (*Mus musculus*) and shrew (*Elephantulus edwardii*) GABPA have amino acid differences in the DNA binding domain; however, based on the available 3D structures of GABPA-DNA interaction, these differences probably do not affect the DNA binding surface of the molecule (Supplementary Fig S2). Consequently, the similarity of different mammalian GABPA proteins suggests that their consensus recognition site is also conserved.

### The presence or absence of a strong GABPA binding site determines TERT expression in somatic cells

**Figure 3:**
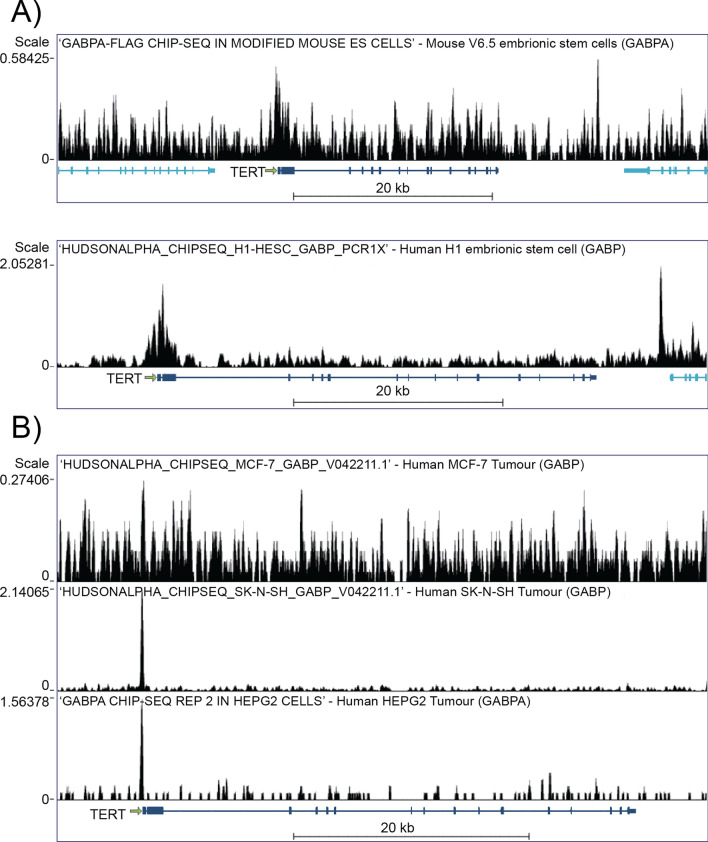
ChIP-Seq data on GABP/GABPA binding to chromatin. (**A**) Publicly available ChIP-Seq data show modest GABPA binding on the TERT promoter of mouse and human embryonic stem cells. (**B**) No GABPA binding is seen on the TERT promoter in either somatic cells or in cancer cell lines without promoter mutation (MCF-7), but in cancer cell lines with TERT promoter mutations (SK-N-SH and HEPG2, C-124T) GABPA binding at the TERT promoter regions is clearly visible.

Next, we searched for more direct proof on the role of GABPA at TERT promoters. For this we used publicly available ChIP-Seq data on GABPA localization and found that both in human and mouse embryonic stem cells the GABP protein complex is present on the TERT promoter (Fig. 3A). On the other hand, in human somatic cells in which the telomerase is inactivated, no GABPA binding is detected. ChIP-Seq data also demonstrate that in SK-N-SH or HEPG2 tumor cells GABPA is present at the TERT promoter region. This is due to consensus binding sites created by point mutations (Fig. 3B). However, in MCF-7 tumor cells no GABPA was detected on the TERT promoter.

**Figure 4:**
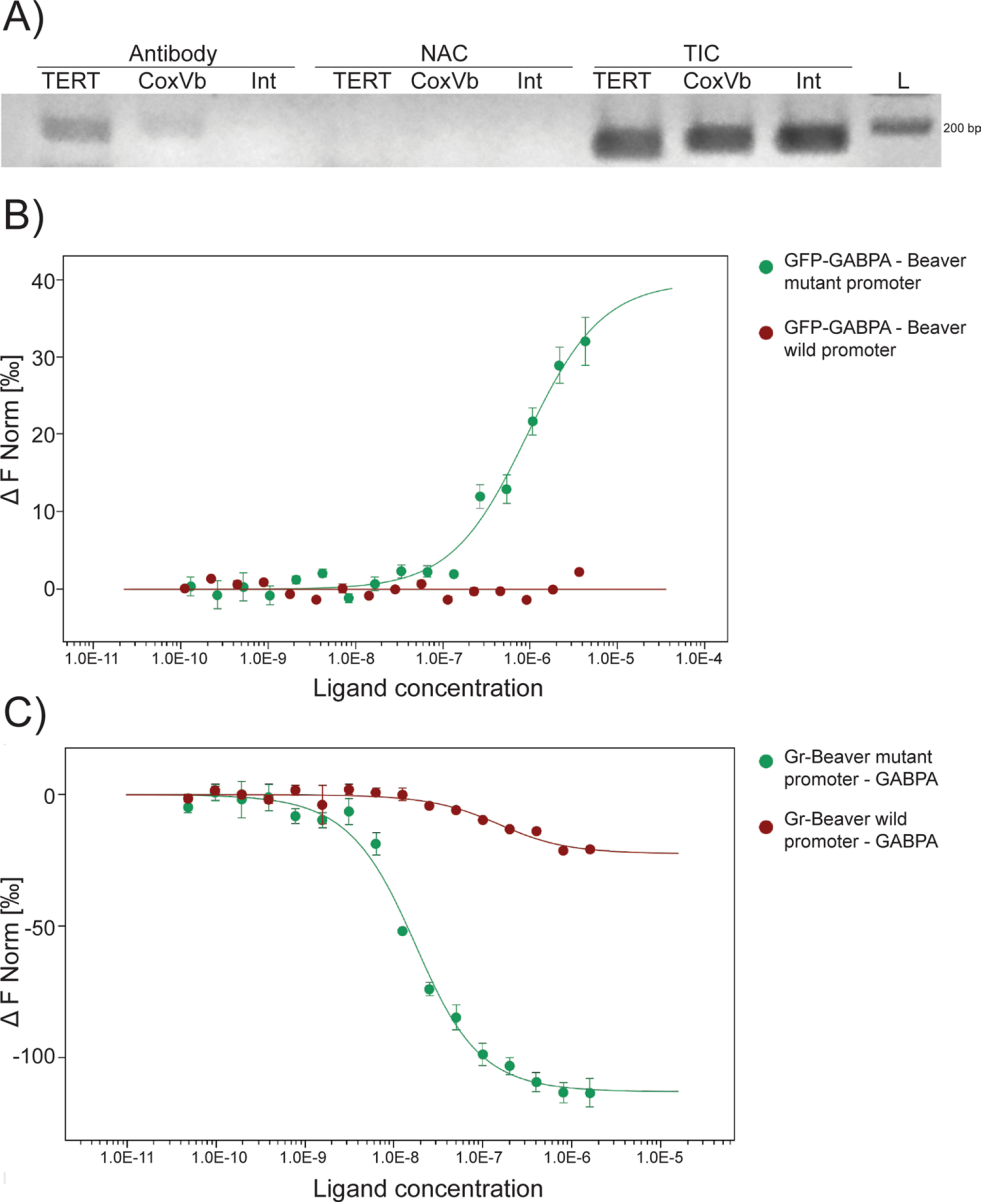
Detection of GABPA binding to mouse and beaver TERT promoters. (**A**) ChIP result indicates GABPA binding to TERT promoter and CoxVb promoter but not to intergenic region in mouse NIH 3T3 fibroblast cells. NAC: no antibody control, TIC: total input control. The plot shows data from one out of three experiments with practically identical results. (**B**,**C**) Microscale thermophoresis (MST) plots indicate GFP-GABPA binding to mutant beaver TERT promoter, but not to wild type beaver promoter. The plots resulted from experiments in which either (**B**) the GABPA protein was fluorescently labelled by GFP fusion, or (**C**) target DNA probes were fluorescently labelled. Standard deviation is calculated from results of technical replicates.

According to our hypothesis, the presence or absence of GABPA binding site at the TERT promoter is the determining factor of telomerase expression in small-bodied rodents such as mice, versus no expression in larger-bodied rodents such as beavers. Among the sequenced mouse genomes accessible at Sanger Centre none carries an SNP that would affect this GABPA binding site, indicating the strong preservation of this motif. To validate that GABPA binding to the TERT promoter of small rodents is indeed a characteristic of not only embryonic stem cells but also of somatic cells, we performed ChIP experiments in the mouse NIH 3T3 cell line, and showed that GABPA binds to the TERT promoter (Fig. 4A). We assume that in beavers (*Castor canadensis*), mutation of the GABPA binding site 150–200 bp upstream of the start codon inactivated TERT expression and allowed replicative senescence to take place. The lack of beaver cell cultures and the difficulties of obtaining biological samples prevented us from conducting ChIP experiments on beaver samples. However, we were able to test whether GABPA is able to bind to the beaver TERT promoter. *Castor canadensis* genomic DNA (kindly provided by Dr. Si Lok) was used to amplify a 500 bp fragment within the TERT promoter region^38^. This promoter fragment harbours a weak GABPA binding site at 164–158 bp upstream of the translation start codon. The score of this site, according to TFbind prediction, is below the cut-off value. We generated a strong GABPA binding site containing a variant of this promoter fragment (with TFbind score prediction above cut-off) by introducing TT > CG changes at position − 159–158 (Supplementary Fig S1). PCR-amplified 500 bp wild type and mutant promoter fragments were used to test their GABPA binding affinity by microscale thermophoresis (MST). For this, GFP-GABPA protein (Supplementary Fig S3) or fluorescently labelled DNA was used. GFP-GABPA showed interaction with the mutant version of the beaver promoter, while no interaction was detected with the wild type promoter (Fig. 4B). The reciprocal experiment with labelled DNA and non-fluorescent GABPA (Supplementary Fig S3) produced similar results (Fig. 4C).

**Figure 5:**
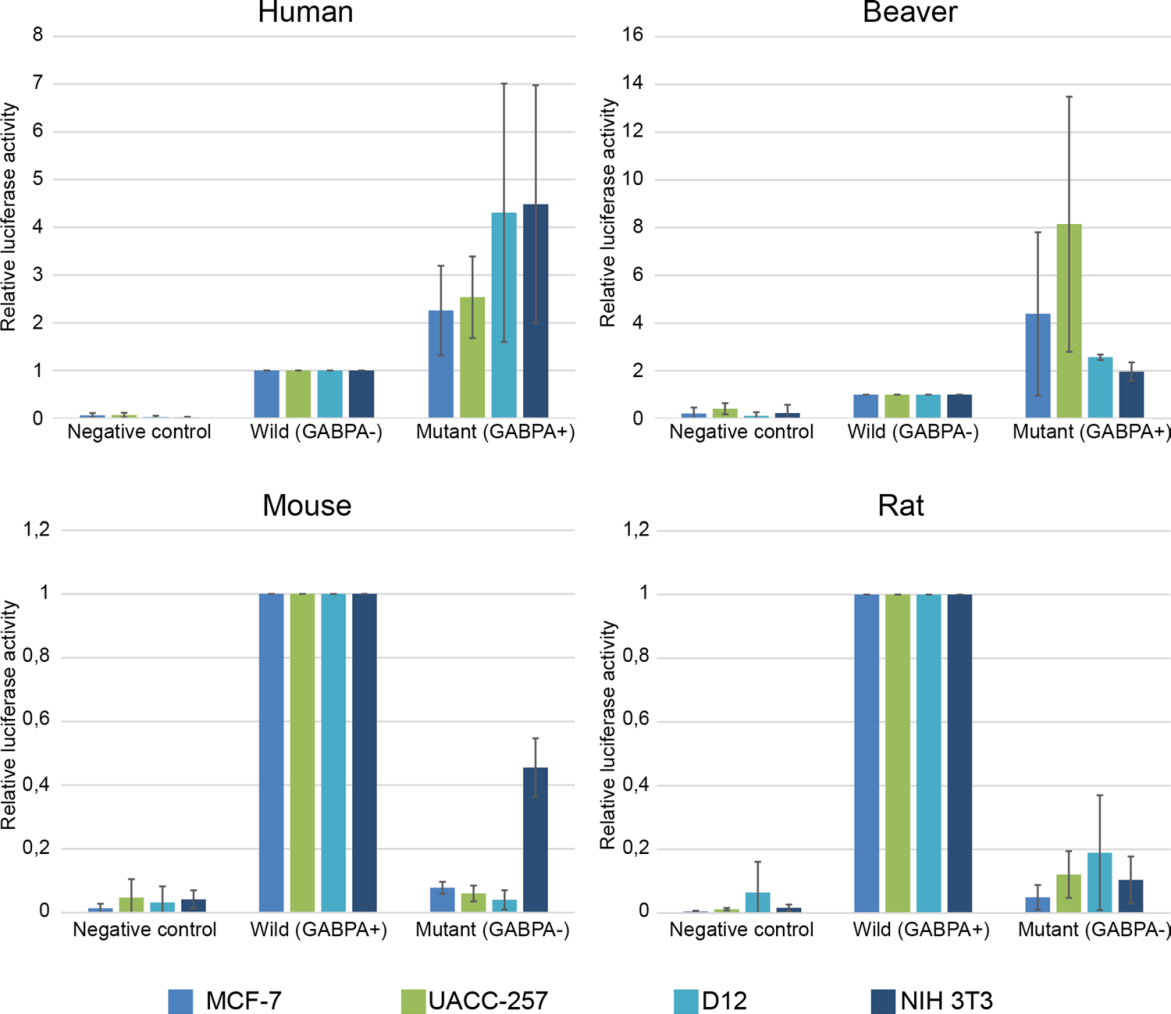
The introduction of the GABPA binding site to human or beaver promoter increased TERT promoter activity, while mutation in the GABPA binding site in mouse or rat TERT promoter greatly decreased promoter activity. Cell lines used in the transient expression assays are indicated at the bottom.

Following these experiments, we intended to test the effect of GABPA binding site on the activity of different TERT promoters. Silencing or knocking out a general transcription factor such as GABPA would most likely disrupt normal cell function, and TERT expression could also be affected as an off-target effect. Instead, we focused specifically on the promoter regions and conducted luciferase reporter assays using human, beaver, rat and mouse TERT promoters in multiple cell lines to obtain conclusive data. We also generated mutant versions of the promoters: a GABPA binding site was introduced into human and beaver promoters (C to T at − 146 and see above, respectively), and GABPA binding sites were removed from the rat and mouse TERT promoters (T to C at − 163 and T to C at − 162, respectively) (for the mutation sites and predicted TFbind scores of the wild type and constructed mutant TERT promoters see Supplementary Fig S1). Wild type and mutant TERT promoter fragments were inserted into pGL3Basic promoter probe vector in front of the luciferase coding sequence, and their activity was tested in two human (MCF-7, breast cancer; UACC-257, melanoma) and two rodent cell lines (D12, rat hepatocarcinoma; NIH 3T3, mouse fibroblast) by transient transfection experiments. Introduction of the GABPA binding site into the human and beaver promoters (GABPA +) increased the activity of these compared to their wild type (GABPA−) counterparts. On the other hand, mutation in the GABPA binding site (GABPA−) greatly decreased the activity of mouse and rat promoters compared to the activity of their wild type (GABPA +) versions (Fig. 5).

## Discussion

Peto’s paradox raises intriguing questions which relate to both species and tumor cell evolution. Several mechanisms have been suggested to resolve the paradox, including inter-species differences in the number of tumor suppressor genes, rate of somatic cell turnover, cell size, efficiency of the immune system and replicative senescence^3,39–43^. However, apart from mathematical models^44–46^, there are limited research data available on the topic^47–52^. Most of the hypotheses offered to resolve Peto’s paradox are based on comparisons made between organisms differing greatly in size, such as comparing whales or elephants with humans. In elephants, for example, the solution for the paradox is in the different number of tumor suppressor genes. However, as DeGregori highlighted, there is no significant difference between human and mouse tumor suppressor mechanisms regarding the number of tumor suppressor genes or even in the effectiveness of the immune system^41^. Nevertheless, a mechanism that can explain the paradox in tumor frequency values between medium- and small-bodied animals should exist. Replicative senescence induced by telomere shortening is an evolved tumor suppressor mechanism in which small-bodied animals differ from medium-sized ones^53^. With the work presented here we intended to seek an explanation for the mechanism of telomerase inactivation, which would also support the tumor suppressor role of telomere shortening in large-bodied rodents^5,8^. With our conclusions we do not question or wish to disregard other mechanisms that might also play a role in tumor suppression.

Studies on the evolution of telomerase agree that telomerase activation/reactivation in somatic cells has taken place several times during the evolution of vertebrates^6,7,22^. According to the calculations of Gomes et al*.* the ancient placental mammalian telomerase was inactive in somatic cells and 5–7 independent events reactivated it during the subsequent evolution of small mammalian species^22^. These independent events suggest that telomerase expression could be beneficial in small animals. Telomerase is known to have other functions in addition to telomere maintenance^54^, as it plays a role in protection against oxidative stress^55^. Since small animals have higher metabolic rates, which increases oxidative stress, telomerase could be beneficial as part of the stress response mechanism. In larger-bodied relatives, however, telomerase expression could be unfavourable due to the necessity of stronger tumor suppression.

Telomerase is a multi-subunit complex; its activity in different placental mammals is determined primarily by the transcription of its catalytic subunit (TERT)^31,56,57^. TERT regulation is quite complex including regulation by transcription factors like the Myc/Max/Mad protein family and many others (Supplementary Fig S4.), miRNAs, alternative splicing, post translational modifications and subcellular trafficking—for reviews see^29,57–61^. The lengths of telomeres in studied species correlate with telomerase expression levels^22^, and several experiments have demonstrated that telomere shortening happens due to the deletion or down-regulation of TERT^24,27^. Sun et al. showed that the loss of one copy of TERT results in sufficient TERT down-regulation for replicative senescence to take place in mice^27^. While enzyme evolution in general means changes in the protein sequence, in the case of telomerase the high frequency of activity changes in somatic cells and the fact that functional telomerase is essential in germ line and stem cells makes this mechanism unlikely, and justifies considering changes at transcriptional level, as seen in other examples^62,63^.

With this prerequisite, we assumed that molecular traces of TERT inactivation and reactivation have remained in the promoters of different species and consequently, comparison of the promoter sequences may unravel such traces. By comparing TERT promoters of 28 species, we found that multiple transcription factor binding motifs showed significant differences in active and inactive promoters, both in respect of their presence or absence and their pattern of location. GABPA (or NRF2), ELK1 and GATA3 transcription factors are more frequent in active promoters, while E47 (or TCF3) factor binding sites are more frequent in inactive promoters. As its binding sites showed the most significant differences between active and inactive promoters, we focused on the GABPA transcription factor. GABPA (NRF2) contributes to mitochondrial biogenesis as a regulator of mitochondrial genes^64^. Surprisingly, under oxidative stress TERT is excluded from the nucleus and transferred to the mitochondria where it plays a protective role against oxidative stress^55^. This links TERT to the regulation of mitochondrial gene expression, and the common regulation strengthens our earlier proposal that active TERT in small animals is beneficial due to its role in oxidative stress reduction. GABPA sites were predicted in similar positions in different species, suggesting its conservation, while recognition sites of the other Ets family member, ELK1, were more dispersed (Fig. 2). A further reason that made the presence or lack of GABPA site fascinating was that de novo GABPA binding motifs that are responsible for telomerase reactivation in human tumors are frequently formed at 124 or 146 bp upstream of the START codon, in similar positions as those sites that we identified in our analysis. Telomerase activation is responsible for the immortalization of approximately 90% of tumor cells. The activation could happen in multiple ways, such as through epigenetic changes, alternative splicing, or by the previously mentioned TERT promoter mutation—for reviews see^31,61^. In tumors the change in the − 124 or − 146 bp position in the promoter regions creates ETS factor recognition motifs. Although the ETS transcription factor family is large (28 members in humans^65^), it was shown that GABPA selectively binds the de novo binding site and activates TERT expression^66,67^. TERT promoter mutations in tumors are very common^56,68,69^, and it is plausible that similar mutations could influence TERT expression during the evolution of rodents. Although GABPA activates TERT in tumors harbouring the promoter mutation, it should not be regarded exclusively as an oncogene. GABPA has tumor suppressor roles as well through the regulation of DICER1, which is responsible for multiple tumor suppressor mechanisms involving the miRNA pathway^70^.

The ETS factor binding site that we identified as a GABPA binding site is missing in beavers, where telomerase is inactivated. Thus, TERT inactivation in beavers could be explained with mutation(s) in a strong GABPA binding site. In the beaver promoter only weak motifs were predicted, while strong binding sites can be found in relatives such as in mice. The experiments performed to validate that GABPA indeed binds to the mice TERT promoter and not to that of beavers supported our assumption: GABPA was detected on the TERT promoter in mouse fibroblast cells, but we could not detect interaction between the transcription factor and beaver TERT promoter. The loss of GABPA binding site, however, does not necessarily mean decreased promoter activity, therefore we performed reporter assays where promoter regions with and without GABPA binding sites were tested. For these functional assays, human and beaver promoters were mutated to introduce GABPA binding motifs. The presence of the binding site increased promoter activity. In contrast, when mutations were introduced to mouse and rat promoters to eliminate GABPA binding sites, the promoter activity was greatly reduced. Considering the experimental proof that links telomerase expression levels to telomere length^27^ and telomere length to the widely accepted tumor suppressor mechanisms of replicative senescence^24^, we conclude that our data support the initial hypothesis. Therefore, one could assume that in the beaver TERT promoter the loss of the GABPA binding motif reduced the promoter activity and inactivated TERT expression in somatic cells, by which replicative senescence was enabled. Since replicative senescence is a powerful tool to keep tumorigenesis at a low level, it is expected to allow beavers to develop larger body mass than other rodents.

It is important to emphasize that the hypothesis that the elimination of GABPA site(s) led to the loss of TERT promoter activity in somatic cells and by this generated replicative senescence as a tumor suppressor mechanism does not exclude the possibility that other effective tumor suppressor mechanisms are active in large-bodied animals. This could be the case in pigs (*Sus scrofa*) and camels (*Camelus dromedarius*), which also have GABPA sites in the TERT promoter and active telomerase^22,71^.

It remains an open question whether apart from GABPA, any of the other sites we identified as being differently represented between active and inactive TERT promoters determined TERT activity during evolution. The E47 helix-loop-helix transcription factor often forms heterodimers with other helix-loop-helix transcription factors like MyoD^72^, a master regulator of muscle development^73^. Indeed, alongside the E47 binding sites we also found significant difference in MyoD binding sites between active and inactive TERT promoters in our random sampling-bootstrap analysis (Supplementary Table S1). However, although the E47 and MyoD heterodimer is an activator of transcription, strong binding sites for them are present in inactive TERT promoters more frequently than in active ones. Therefore, we do not presume that these elements play a role in telomerase silencing in somatic cells. However, they might have a role in maintaining sufficient telomerase expression in stem cells after the loss of ubiquitous expression of TERT.

The GATA3 transcription factor could also have a significant role in telomerase regulation, especially in group IV (Fig. 1A). Active telomerase and strong GATA3 binding motifs can be observed in polecats and bats, but also in the TERT promoter of camels and pigs, which are some of the rare exceptions of having active telomerase despite large body size. These motifs are missing in sheep (*Ovis aries*) and rhinoceros (*Ceratotherium simum sinum*), which are their closest studied relatives (Fig. 1B)^7,22^. GATA3 expression levels are not uniform in all tissues; it has low expression levels in the brain, gut, liver and pancreas in humans and mice and expectedly in other mammals (Supplementary Table S4)^74,75^, therefore the absence or presence of GATA3 sites probably has no effect in these tissues. On the other hand, GATA3 expression levels are higher in fibroblasts from skin, kidney, lung or cornea, in which tissues telomerase activity was also tested and found active^22^. Based on these observations a hypothesis can be formed that may explain the telomerase activity in bats, polecats, camels and pigs, which remains speculative until experimentally tested.

## Materials and methods

### Collection of data

TERT promoter and GABPA transcription factor sequences were downloaded from NCBI database. Identifiers are summarized in Supplementary Table S5. Data on telomerase activity in different species were obtained from the literature^6,7,22,32–34^. 3D structure of GABPA DNA binding domain was recovered from PDB database (1AWC)^76^. ChIP-Seq data were obtained from available databases (Supplementary Table S6).

### Bioinformatics

Transcription factor binding sites were determined by Match Publicversion 1.0 (TRANSFAC Public 7.0 ) and TFbind software (TRANSFAC R.3.4 database)^35^. Match uses Transfac database containing 6133 factors. Transfac R.3.4 used by TFbind contains ~ 8000 binding sites. In humans, 1639 transcription factors are present from which 1107 have experimentally measured binding sites^77,78^. Since both Match and TFbind use relatively old databases, after prediction we compared the results to known transcription factor binding sites on human promoters^29,57,58^ (Supplementary Fig S4). Both Match and TFbind use manually set cut-off values to filter data. For our analysis, we set strict cut off values: in case of Match we used 1.0 core similarity and 0.94 overall matrices similarity; in case of TFbind 0.9 was set as cut off.

The number of samples we could use naturally was limited by the availability of data on TERT promoter sequences and telomerase activity among mammals. We therefore selected statistical methods suitable for small sample sizes to compare active and inactive promoters (Random sampling bootstrapping, Fisher’s exact test). For Random sampling bootstrapping we used R, while two tailed Fisher’s exact tests were done by GraphPad [https://www.graphpad.com/quickcalcs/contingency1.cfm]. We used FIMO and Jaspar2020 database to calculate p-values for GABPA, Elk1, E47,GATA3 transcription factor binding sites identified by TFbind or Match (Supplementary Table S8)^79^. We also compared the methods of TF binding site prediction focusing on GABPA (Supplementary Fig S5). K-means clustering was done in Python3.6.3 using the KMeans algorithm from the scikit-learn library. To visualize GABPA 3D structure, UCSF Chimera was used^80^. Masses of the examined species were obtained from PanTHERIA database^81^. Canis familiaris weight is between 1.36 (Pomeranian) and 104.32 kg (Mastiffs) according to the American Kennel Club [https://www.akc.org/expert-advice/nutrition/breed-weight-chart/]; we presented the mass of an average German shepherd. Mouse Tert promoter sequences were extracted from Mouse Genomes Project data [https://www.sanger.ac.uk/science/data/mouse-genomes-project]^82^. Scripts used are available in Supplementary Note S1.

### ChIP

Chromatin was cross-linked and nuclei were isolated using cell lysis buffer (5 mM PIPES pH 8.0, 85 mM KCl, 0.5% NP40, 1 × PIC). Nuclei were lysed in nuclear lysis buffer (50 mM TRIS–HCl pH 8.0, 10 mM EDTA pH 8.0, 0.8% SDS, 1 × PIC), and sonicated in Bioruptor sonicator. 20–30 μg of total chromatin diluted in dilution buffer (10 mM TRIS pH 8.0, 0.5 mM EGTA pH 8.0, 1% Triton X-100, 140 mM NaCl, 1 × PIC) to 500 μl was used for each sample. Antibody (Ab) and no antibody (NAC) samples were pre-cleared with Dynabeads M-280 Sheep Anti-Rabbit IgG (Thermo Fisher Scientific, Waltham, Massachusetts, USA). 5 μg of primary GABPA Antibody (GABPA Polyclonal Antibody, Thermo Fisher Scientific, AB_2545211) was used in the Ab sample. Following incubation (1 h 4 °C), 40 μl Dynabeads were added. In the total input control (TIC) no antibodies or magnetic beads were used. After incubation (2 h, 4 °C), washing steps were done using freshly prepared Low Salt Wash Buffer (0.1% SDS, 1% triton X-100, 2 mM EDTA, 20 mM Tris–HCl pH 8.0, 150 mM NaCl), High Salt Wash Buffer (0.1% SDS, 1% triton X-100, 2 mM EDTA, 20 mM Tris–HCl pH 8.0, 500 mM NaCl), LiCl Wash Buffer (0.25 M LiCl, 1% NP-40, 1% Sodium Deoxycholate, 1 mM EDTA, 10 mM Tris–HCl pH 8.0) and TE Buffer (10 mM Tris–HCl pH 8.0, 1 mM EDTA) at 4 °C. DNA was eluted with elution buffer (1% SDS, 100 nM NaHCO3). The eluted samples were reverse cross-linked, RNase and proteinase treated, and DNA was purified with phenol-chlorophorm-isoamylalcohol (25:24:1) extraction. The regions of interest were amplified with PCR. Primers are listed in Supplementary Table S7.

### Cloning

Cloning was done by HiFi assembly (NEB, Ipswich, Massachusetts, US, E5520S). DNA fragments were amplified with Q5 polymerase (NEB, M0491S). The list of primer sequences used for cloning different desired fragments can be found in Supplementary Table S7. For reporter assay constructs pGL3b, for protein expression pET16b vectors were used as backbones. The mutant promoter sequences were produced by PCR mutagenesis and verified by Sanger sequencing. Primers are listed in Supplementary Table S7.

### Protein production and purification

GABPA proteins were produced in the Six Pack *E. coli* cell line^83^. Cells were suspended in buffer A (20 mM HEPES pH 8.0, 50 mM imidazole, 400 mM NaCl) supplemented with proteinase inhibitor cocktail (PIC), and sonicated. The cell extracts were loaded on Nickel-NTA agarose column (QiaGen, Hilden, Germany) and after washing, heterologous proteins were eluted in buffer B (20 mM HEPES pH 8.0, 250 mM imidazole, 400 mM NaCl). Samples were concentrated and the buffer was changed using Amicon ultra filter units (Merck, Darmstadt, Germany).

### Microscale thermophoresis

This technique is suitable to detect macromolecule interactions based on the changes in thermophoretic properties of macromolecules. The assay requires the fluorescent labelling of one of the interacting partners. For this we used either GFP-GABPA or GrGreen DNA dye (Lab Supply Mall Inno Vita Inc. Beijing, China). 16 step dilution series were prepared using wild type and mutant beaver promoter fragments from 3.36 mM and 4.3 mM starting concentrations, respectively. GFP-GABPA (20 nM concentration) and DNA samples were mixed and incubated at room temperature for 20 min. For the reciprocal experiment, wild type and mutant beaver TERT promoter DNA fragments dyed by GrGreen DNA dye and purified his-GABPA protein (in concentrations from 1,6 uM to 48,9 pM in a16 step dilution series) were mixed. The measurements were done in buffer containing 20 mM TRIS pH 8.0, 50 mM NaCl, 10% Glycerol, 0.05% NP40, in standard capillaries. The equipment used was Monolith NT 115 (Nanotemper, München, Germany).

### Cell culture

The dexamethasone-resistant hepatoma rat cell line (D12) was isolated and described by Venetianer et al.^84,85^. Cell lines NIH 3T3 and MCF-7 were obtained originally from ATCC (Manassas, Virginia, USA, CRL-1658, HTB-22). UACC-257 cell line is from cancer panel NCI-60. Each cell line was maintained and used in tissue culture laboratories regularly at our Department. Mycoplasma infection of the cell lines was tested regularly using MycoAlert Mycoplasma Detection Kit (Lonza, Basel, Switzerland, LT07-418) as described by the supplier. Each cell line used here was free of detectable mycoplasma infection at the time used in the experiments described here. Cell lines were grown in Ham’s F12 (D12), RPMI (MCF-7, UACC-257), and DMEM (NIH3T3) media, supplemented with 10% fetal bovine serum (5% for Ham’s F12), 2 mM of glutamine and antibiotics.

### Reporter assays

D12, MCF-7, UACC-257 and NIH 3T3 cell lines used for transient transfection followed by luciferase reporter assay. Transfections were done using Turbofect reagent (Thermo Scientific). For this 2.5 × 10^5^ cells were seeded into 6 well cell culturing plates. 4 μg of plasmid DNA was measured to 400 μl of serum free media, then 6 μl of transfection reagent was added to the solution and mixed by pipetting. After 20 min of incubation at room temperature the mixture was pipetted on the cells. The plate was gently rocked to mix the solution. Cells were collected 24 h after transfection using cell scratchers. Luciferase expression levels were determined using Luciferase Assay system (Promega, Madison, WI USA) as has been described^85^. The measurements were performed in Orion L type Microplate Luminometer (Berthold Detection System, Simplicity 4.2 software). The protein concentrations were determined by Bradford reaction and the measured expression values were normalized using this data.

All experiments were conducted in at least 3 biological replicates. Outliers within these experiments were not excluded.

## Supplementary information

Supplementary information.

## Data Availability

Data analysed during this study are included in this published article (and its supplementary information files). The datasets used and/or analysed during the current study are available from the corresponding author on reasonable request.
