# Supplementary material for: TERT promoter alterations could provide a solution for Peto’s paradox in rodents
Source: Sci Rep. 2020 Nov 30;10:20815. doi: 10.1038/s41598-020-77648-0 (PMC7704627; doi:10.1038/s41598-020-77648-0)
Supplement: Supplementary file 1 — Supplementary information. [file 41598_2020_77648_MOESM1_ESM.pdf]

TERT promoter alterations could provide a solution for Peto's paradox in rodents

Balázs Vedelek[1,2], Asha Kiran Maddali[1,3], Nurgul Davenova[1], Viktor Vedelek[4], Imre M. Boros[1,2,#]

[1]Department of Biochemistry and Molecular Biology, University of Szeged, Szeged, Hungary

[2]Institute of Biochemistry, Biological Research Centre, Szeged, Hungary

[3]Institute of Genetics, Biological Research Centre, Szeged, Hungary

[4]Department of Genetics, University of Szeged, Szeged, Hungary

[#] Corresponding author borosi@bio.u-szeged.hu

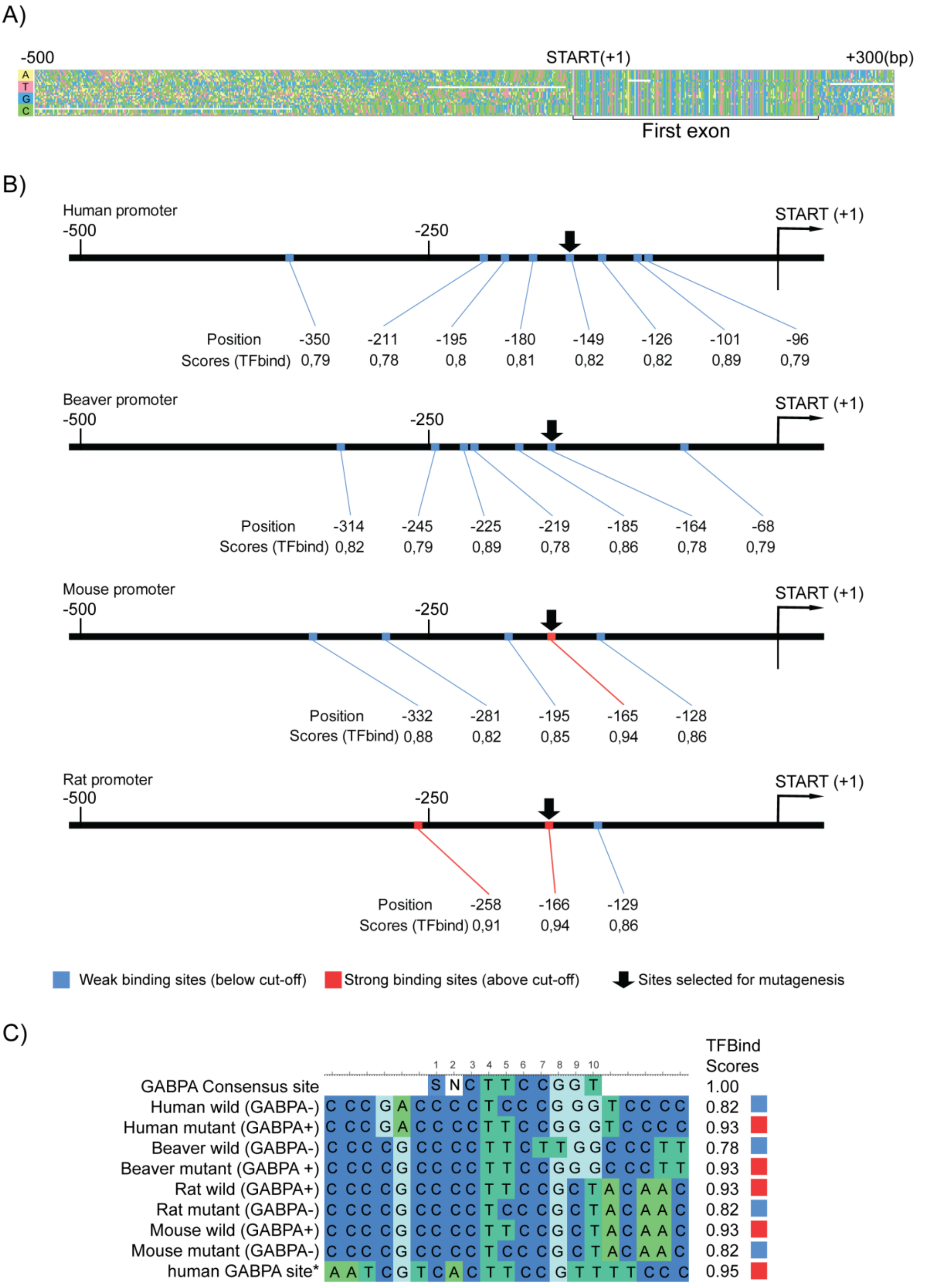

Supplementary Fig S1 GABPA binding sites selected for mutagenesis. (A) The promoter regions of the TERT genes are less conserved than the coding regions. However, similarities among more closely

related species can be observed. (B) There is no GABPA binding to TERT promoter in human somatic cells, therefore we suppose there are only "weaker" binding sites (below cut off value - blue) present on the TERT promoter. A similar distribution of predicted sites is present on the beaver TERT promoter.

However, in case of mouse and rat we find "stronger" binding sites (above the cut of value - red). For mutagenesis we selected the binding sites close to -150 upstream of the start codon. (C) Multiple sequence alignment of the wild type and mutant GABPA sites. Asterisk marks the sequence used by Suzuki et al. to study GABPA binding *in vitro* [1]

Supplementary Table S1 Comparison of the most abundant transcription factor binding matrices between active and inactive TERT promoters by random sampling-bootstrap method.

Factors	Vertebrata matrices (samples 1-60)	Prediction method	inactive>active (p-value)	inactive=active (p-value)	inactive<active (p-value)	Sum -Difference from 1[a]
NRF2 (GABPA)	V$NRF2_01	TFBind	0,0000	0,0272	0,9710	-0,0018
NRF2 (GABPA)	V$NRF2_01	MATCH	0,0000	0,0834	0,9172	0,0006
USF	V$USF_01	MATCH	0,0006	0,7428	0,2626	0,0060
GATA3	V$GATA3_01	TFBind	0,0008	0,2022	0,7892	-0,0078
USF	V$USF_C	MATCH	0,0008	0,7852	0,2116	-0,0024
ELK1	V$ELK1_02	MATCH	0,0012	0,0906	0,9078	-0,0004
USF	V$USF_02	TFBind	0,0012	0,9234	0,0796	0,0042
NMYC	V$NMYC_01	MATCH	0,0016	0,7406	0,2530	-0,0048
CETS1-P54	V$CETS1P54_02	TFBind	0,0020	0,6094	0,3968	0,0082
ARNT	V$ARNT_01	MATCH	0,0022	0,6418	0,3628	0,0068
CEBPB	V$CEBPB_01	TFBind	0,0096	0,6066	0,3782	-0,0056
CDXA	V$CDXA_01	TFBind	0,0116	0,0502	0,9442	0,0060
VMYB	V$VMYB_02	MATCH	0,0140	0,7852	0,1992	-0,0016
PBX1	V$PBX1_01	TFBind	0,0154	0,7908	0,2076	0,0138
MAX	V$MAX_01	MATCH	0,0174	0,9148	0,0732	0,0054
MYB	V$MYB_Q6	TFBind	0,0280	0,2148	0,7626	0,0054
MZF1	V$MZF1_02	TFBind	0,0368	0,3922	0,5844	0,0134
CAP	V$CAP_01	TFBind	0,0378	0,0266	0,9360	0,0004
MYC-MAX	V$MYCMAX_02	TFBind	0,0436	0,5876	0,3756	0,0068
CETS1-P54	V$CETS1P54_01	MATCH	0,0478	0,5762	0,3690	-0,0070
CP2	V$CP2_01	TFBind	0,0528	0,2090	0,7472	0,0090
VMYB	V$VMYB_02	TFBind	0,0668	0,8672	0,0672	0,0012
CETS1-P54	V$CETS1P54_01	TFBind	0,0708	0,3022	0,6220	-0,0050
IK2	V$IK2_01	TFBind	0,0974	0,2914	0,6118	0,0006
GATA2	V$GATA2_01	TFBind	0,0984	0,2928	0,6024	-0,0064
NMYC	V$NMYC_01	TFBind	0,1016	0,2880	0,6198	0,0094
USF	V$USF_C	TFBind	0,1340	0,1622	0,6912	-0,0126
IK1	V$IK1_01	MATCH	0,1706	0,6650	0,1690	0,0046
CEBP	V$CEBP_01	TFBind	0,1850	0,3474	0,4464	-0,0212
NF-KAPPAB	V$NFKAPPAB50_01	TFBind	0,1946	0,7944	0,0028	-0,0082
AP1	V$AP1_Q2	MATCH	0,2048	0,3598	0,4476	0,0122
SP1	V$SP1_01	TFBind	0,2138	0,2090	0,5710	-0,0062
MZF1	V$MZF1_01	TFBind	0,2344	0,2860	0,4650	-0,0146
PADS	V$PADS_C	TFBind	0,2772	0,4554	0,2766	0,0092
HSF2	V$HSF2_01	TFBind	0,2816	0,4580	0,2760	0,0156
MYC-MAX	V$MYCMAX_02	MATCH	0,2834	0,4366	0,2828	0,0028
GATA1	V$GATA1_01	TFBind	0,3176	0,3866	0,2844	-0,0114
P53	V$P53_02	TFBind	0,3278	0,5354	0,1236	-0,0132
SRY	V$SRY_01	TFBind	0,3340	0,1790	0,4954	0,0084
AP4	V$AP4_Q6	TFBind	0,3608	0,2718	0,3656	-0,0018

DELTAEF1	V$DELTAEF1_01	TFBind	0,3752	0,4280	0,1934	-0,0034
AREB6	V$AREB6_01	MATCH	0,3758	0,5614	0,0484	-0,0144
AML1	V$AML1_01	TFBind	0,3852	0,4216	0,1912	-0,0020
CREL	V$CREL_01	TFBind	0,4364	0,3542	0,2152	0,0058
MYOD	V$MYOD_01	TFBind	0,5560	0,3340	0,0988	-0,0112
LMO2COM	V$LMO2COM_01	TFBind	0,5816	0,2540	0,1488	-0,0156
AP1	V$AP1_Q4	TFBind	0,5986	0,3888	0,0068	-0,0058
E47	V$E47_01	MATCH	0,6024	0,3866	0,0008	-0,0102
LYF1	V$LYF1_01	TFBind	0,6066	0,3512	0,0378	-0,0044
AP4	V$AP4_Q5	TFBind	0,6286	0,1922	0,1764	-0,0028
AREB6	V$AREB6_02	MATCH	0,6888	0,2088	0,1110	0,0086
E47	V$E47_02	MATCH	0,7134	0,2270	0,0586	-0,0010
AREB6	V$AREB6_03	MATCH	0,7464	0,1586	0,0808	-0,0142
GC	V$GC_01	TFBind	0,7510	0,2368	0,0106	-0,0016
NF-KAPPAB	V$NFKAPPAB_01	TFBind	0,7782	0,2098	0,0038	-0,0082
USF	V$USF_Q6	TFBind	0,7930	0,1030	0,0854	-0,0186
SP1	V$SP1_Q6	TFBind	0,8492	0,1314	0,0222	0,0028
MYOD	V$MYOD_Q6	TFBind	0,8882	0,0740	0,0338	-0,0040
AP2	V$AP2_Q6	TFBind	0,8960	0,0428	0,0652	0,0040
E47	V$E47_01	TFBind	0,9136	0,0886	0,0000	0,0022
					avg.	-0,0013
					dev.	0,0086

Three hypotheses (whether the binding site is more frequent in active or in inactive promoter or there is no difference between them) were tested by random sampling bootstrap method in independent runs.

Random sampling bootstrapping is suitable for testing hypotheses. In our case to test whether a transcription factor binding site is more or less frequent in active or inactive TERT promoters. Bootstrapping was our choice of technique given the limited sample size due to available data on telomerase status and telomere sequence in mammals. Using the available transcription factor binding site frequency data, we performed random sampling and comparisons 5000 times. From this approach we obtained probability values. Comparisons were done to check whether the mean is equal, smaller or larger between samples. In an ideal case the sum of probabilities from the three independent runs would be one. The difference from 1 can be interpreted as the error rate of the method.

Supplementary Table S2 Comparison of rarer transcription binding matrices between active and inactive TERT promoters by Fisher's exact test.

Factor	Vertebrata matrices (samples 26-97)	Prediction method	Fisher's exact test (p-value two-tailed)
NRF2 (GABPA)	V$NRF2_01	MATCH	0,0002
E47	V$E47_01	TFBind	0,0183
NRF2	V$NRF2_01	TFBind	0,0213
TST1	V$TST1_01	TFBind	0,0407
ELK1	V$ELK1_02	TFBind	0,0407
GATA3	V$GATA3_01	TFBind	0,0461
ELK1	V$ELK1_02	MATCH	0,0542
ZID	V$ZID_01	TFBind	0,0768
E2F	V$E2F_02	TFBind	0,0978
E47	V$E47_01	MATCH	0,1032
NF-KAPPAB	V$NFKAPPAB_01	TFBind	0,1201
USF	V$USF_Q6	TFBind	0,1283
CEBPB	V$CEBPB_02	TFBind	0,1647
CETS1-P54	V$CETS1P54_02	TFBind	0,2087
AP1	V$AP1_Q4	TFBind	0,2365
MYB	V$MYB_Q6	TFBind	0,2519
P300	V$P300_01	TFBind	0,3259
AHRARNT	V$AHRARNT_01	MATCH	0,3259
NF-KAPPAB 50	V$NFKAPPAB50_01	TFBind	0,3845
OCT1	V$OCT1_03	TFBind	0,3845
CEBPB	V$CEBPB_01	TFBind	0,4197
MYOD	V$MYOD_01	MATCH	0,4197
MZF1	V$MZF1_02	TFBind	0,4401
E47	V$E47_02	MATCH	0,4495
STAT	V$STAT_01	TFBind	0,4815
OCT1	V$OCT1_06	TFBind	0,5956
S8	V$S8_01	MATCH	0,5956
HSF1	V$HSF1_01	TFBind	0,5956
CREBP1-CJUN	V$CREBP1CJUN_01	TFBind	0,5956
AREB6	V$AREB6_03	MATCH	0,6483
USF	V$USF_02	TFBind	0,6483
NKX25	V$NKX25_01	MATCH	0,6483
PADS	V$PADS_C	MATCH	0,6483
HAND1-E47	V$HAND1E47_01	MATCH	0,6483
PBX1	V$PBX1_01	TFBind	0,6776
VMYB	V$VMYB_02	MATCH	0,6776
AP1	V$AP1_Q2	TFBind	0,6776

AREB6	V$AREB6_01	MATCH	0,6946
CETS1-P54	V$CETS1P54_01	MATCH	0,6946
MYC-MAX	V$MYCMAX_02	TFBind	0,6946
IK2	V$IK2_01	TFBind	0,7036
MYOD	V$MYOD_01	TFBind	0,7036
CETS1-P54	V$CETS1P54_01	TFBind	1,0000
P53	V$P53_02	TFBind	1,0000
CREL	V$CREL_01	TFBind	1,0000
CEBP	V$CEBP_01	TFBind	1,0000
PADS	V$PADS_C	TFBind	1,0000
MYC-MAX	V$MYCMAX_02	MATCH	1,0000
HSF2	V$HSF2_01	TFBind	1,0000
AP1	V$AP1_Q2	MATCH	1,0000
IK1	V$IK1_01	MATCH	1,0000
MAX	V$MAX_01	MATCH	1,0000
VMYB	V$VMYB_02	TFBind	1,0000
AP1	V$AP1_C	TFBind	1,0000
AP1FJ	V$AP1FJ_Q2	TFBind	1,0000
NF-KAPPAB	V$NFKAPPAB65_01	TFBind	1,0000
VMYB	V$VMYB_01	MATCH	1,0000
USF	V$USF_Q6	MATCH	1,0000
AP1	V$AP1_Q4	MATCH	1,0000
NKX25	V$NKX25_01	TFBind	1,0000
CDPCR3HD	V$CDPCR3HD_01	TFBind	1,0000
HLF	V$HLF_01	TFBind	1,0000
NGFIC	V$NGFIC_01	TFBind	1,0000
NF-KAPPAB	V$NFKB_Q6	TFBind	1,0000
ATF	V$ATF_01	MATCH	1,0000
RORA1	V$RORA1_01	MATCH	1,0000
HNF3B	V$HNF3B_01	TFBind	1,0000
CDXA	V$CDXA_02	TFBind	1,0000
AP1	V$AP1_Q6	TFBind	1,0000
HFH2	V$HFH2_01	TFBind	1,0000
MAX	V$MAX_01	TFBind	1,0000
USF	V$USF_01	TFBind	1,0000

Fisher's exact test is employed to analyse contingency tables of categorical variables. Though it is independent of sample size, it is generally used for small samples. We employed two tailed Fisher's exact tests on those cases where the transcription factor frequency turned out to be a categorical variable (present or absent). We created contingency tables that depict active or inactive telomeres and the presence or absence of the transcription factor. This test provided exact p-values listed in Supplementary 3

Supplementary Table S3 Clusters that could be formed based on the occurrence of transcription factors and telomerase activity.

Latin name	K-1	K-2	K-3	K-4	K-5	K-6	K-7	K-8	K-9	K-10	K-11	K-12	K-13	K-14	K-15	K-16	K-17	K-18	K-19	K-20
Bos taurus	0	1	1	2	0	0	0	2	0	0	1	1	1	3	2	0	1	1	0	3
Castor canadensis	0	1	1	2	0	1	1	1	4	4	3	1	1	1	3	0	1	1	4	1
Eschrichtius robustus	0	1	1	2	0	1	1	1	0	0	1	1	1	3	2	0	1	1	0	3
Tursiops truncates	0	1	1	2	0	1	1	1	0	0	1	1	1	3	2	0	1	1	0	3
Canis familiaris	0	1	1	2	0	2	2	3	0	0	1	1	1	3	2	0	1	1	0	3
Equus caballus	0	1	1	2	0	2	2	3	0	0	1	1	1	3	2	0	1	1	0	3
Ovis aries	0	1	1	2	0	2	2	3	4	4	3	1	1	1	3	0	1	1	4	1
Mus musculus	1	0	0	3	2	0	0	2	1	1	0	0	0	0	1	4	4	2	2	0
Elephantulus edwardii	1	0	0	3	2	1	1	1	1	1	0	0	0	0	1	4	4	2	2	0
Mesocricetus auratus	1	0	0	3	2	1	1	1	1	1	0	0	0	0	1	3	0	0	2	2
Peromyscus maniculatus bairdii	1	0	0	3	2	1	1	1	1	1	0	0	0	0	1	3	0	0	2	2
Rattus norvegicus	1	0	0	3	2	1	1	1	1	1	0	0	0	0	1	4	4	2	2	0
Cavia porcellus	1	0	4	1	1	1	1	1	2	3	4	4	2	0	1	1	0	2	2	0
Heterocephalus glaber	1	0	4	1	1	4	3	4	2	3	4	4	2	0	1	1	0	2	2	0
Sus scrofa	1	0	4	1	1	4	3	4	2	3	4	4	2	0	1	1	0	2	2	0
Microcebus murinus	1	0	4	1	1	3	4	0	2	3	4	4	2	0	1	1	0	2	2	0
Giraffa camelopardalis tippelskirchi	2	2	0	3	2	1	1	1	1	1	0	0	0	4	0	3	3	4	1	2
Loxodonta africana	2	2	2	0	3	1	1	1	3	2	2	3	4	4	0	2	3	4	1	1
Camelus dromedaries	3	3	2	0	3	1	1	1	3	2	2	2	3	2	4	2	2	3	3	4
Myotis lucifugus	3	3	2	0	3	2	2	3	3	2	2	2	3	2	4	2	2	3	3	4
Ochotona princeps	3	3	2	0	3	2	2	3	3	2	2	3	4	1	3	2	2	3	3	1
Eptesicus fuscus	3	3	2	0	3	4	3	4	3	2	2	2	3	2	4	2	2	3	3	4
Mustela putorius eversmannii	3	3	2	0	3	4	3	4	3	2	2	2	3	2	4	2	2	3	3	4
Ceratotherium simum simum	4	4	3	4	4	0	0	2	0	0	1	1	1	3	2	0	1	1	0	3
Homo sapiens	4	4	3	4	4	0	0	2	4	4	3	3	4	1	3	0	1	1	4	1
Papio anubis	4	4	3	4	4	1	1	1	0	0	1	1	1	3	2	0	1	1	0	3
Macaca mulatta	4	4	3	4	4	2	2	3	0	0	1	1	1	3	2	0	1	1	0	3
Pongo pygmaeus	4	4	3	4	4	3	4	0	4	4	3	3	4	1	3	0	1	1	4	1

A)

Scientific name	Common name	Dissimilarity full length protein	Disimilarity DNA binding domain (321-428. aa)	Refseq/uniprot accession number
Tursiops truncatus	Common bottlenose dolphin	1%	0%	XP_004318161.1
Bos taurus	Cattle	1%	0%	Q0VC98
Ovis aries	Sheep	2%	0%	AAC39262.1
Sus scrofa	Pig	2%	0%	XP_020927100.1
Camelus dromedarius	Dromedary camel	2%	0%	XP_010975695.1
Equus caballus	Horse	2%	0%	XP_014591809.1
Canis lupus familiaris	Dog	1%	0%	XP_013965264.1
Mustela putorius furo	Domestic ferret	1%	0%	XP_004757497.1
Eptesicus fuscus	Big brown bat	2%*	0%*	XP_008143833.1
Myotis lucifugus	Small brown bat	3%	0%	XM_014461936.1
Mus musculus	House mouse	4%	2%	Q00422
Rattus norvegicus	Brown rat	2%	0%	D4ACQ9
Peromyscus maniculatus bairdii	Deer mouse	2%	0%	XP_015861914.1
Castor canadensis	American beaver	1%	0%	JAV37339.1
Heterocephalus glaber	Naked mole-rat	1%	0%	XP_004842281.1
Cavia porcellus	Guinea pig	2%	0%	XP_013005230.1
Mesocricetus auratus	Golden hamster	2%	0%	XP_012971658.1
Ochotona princeps	American pika	2%	0%	XP_004588659.1
Macaca mulatta	Rhesus macaque	0%	0%	F7E124
Pongo abelii	Sumatran orangutan	0%	0%	XP_009232128.1
Papio anubis	Olive baboon	0%	0%	XP_003895635.1
Elephantulus edwardii	Cape elephant shrew	2%	1%	XP_006896098.1
Loxodonta africana	African elephant	2%	0%	XP_003410373.1
Homo sapiens	Human	-	-	Q06546
Ornithorhynchus anatinus	Duck-billed platypus	7%	0%	XP_028938340.1
Oncorhynchus mykiss	Rainbow trout	25%	4%	XP_021453533.1

*partially missing sequence

B)

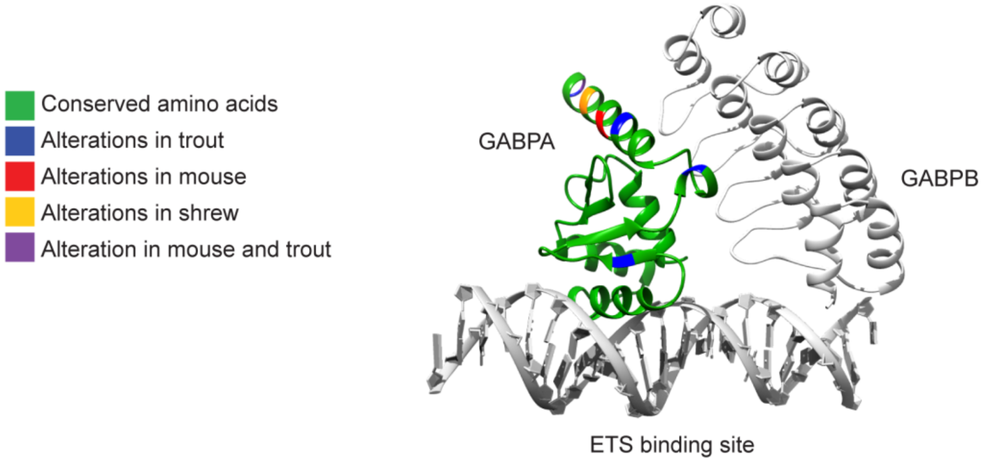

Supplementary Fig S2 GABPA is highly conserved among mammals. (A) Protein sequence differences compared to human GABPA protein show low diversity, with identical DNA binding domain in most cases. (B) 3D structure of GABPA and GABPB. The differences from human GABPA are highlighted for each relevant species.

A)

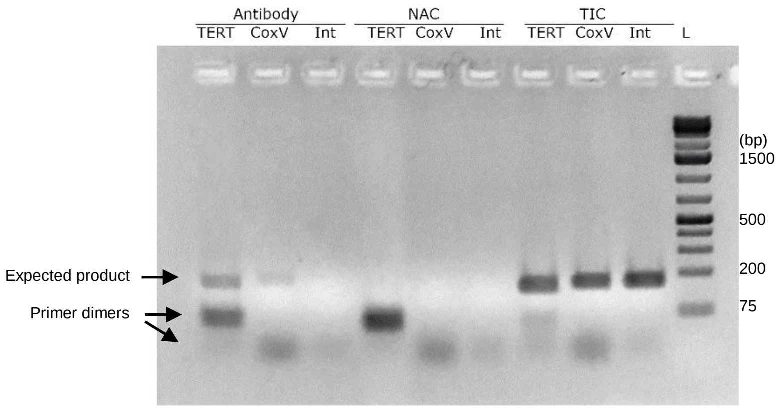

B)

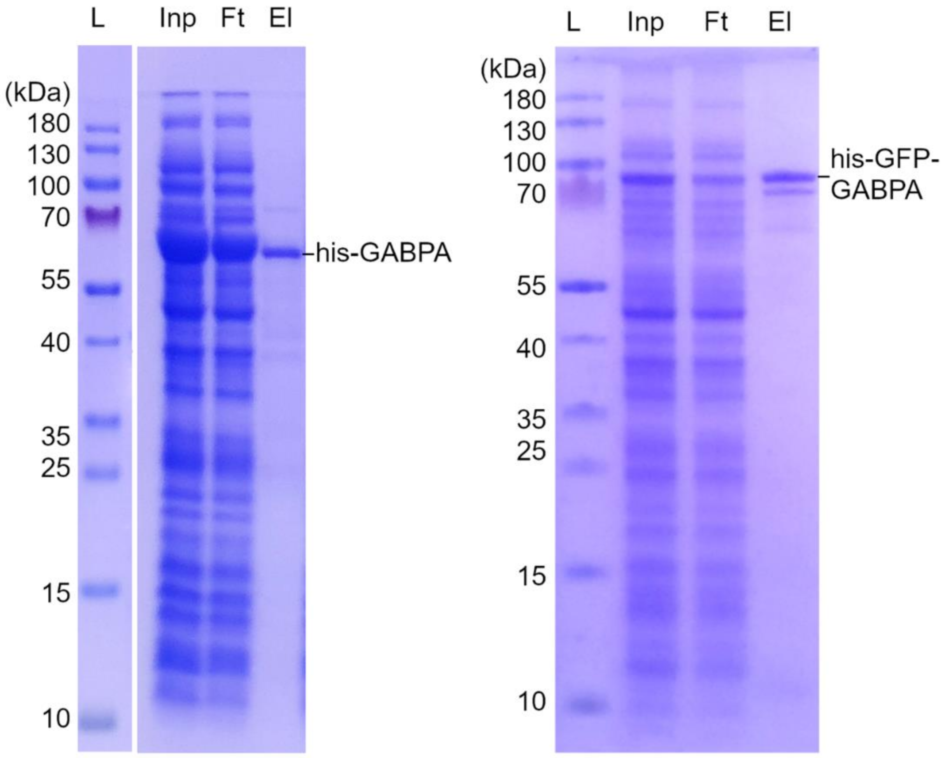

Supplementary Fig S3 (A) Uncropped version of Fig. 4A. (B) Coomasie stained acrylamide gel shows his-GABPA and his-GFP-GABPA, purified from Six Pack cells on Ni-NTA column.

Supplementary Table S4 Gene expression levels of the identified transcription factors in different tissues of human and mouse.

#BioProject
PRJEB4337
Human

		RPKM												
#GeneID	TF	adrenal	appendix	bone marrow	brain	colon	duodenum	endometrium	esophagus	fat	gall bladder	heart	kidney	liver
2625	GATA3	3,15	2,02	1,4	0,0153	0,383	0,423	0,317	0,263	4,5	0,717	0,342	13,3	0,418
2551	GABPA	8,81	9,19	6,08	6,01	8,05	6,71	11,3	6,65	10,7	9,41	5,39	7,08	4,77
2002	Elk1	11,7	6,74	5,03	15,9	5,75	3,72	7,13	4,4	5,66	6,59	5,07	4,86	3,26
6929	E47	1,89	8,4	9,8	3,65	6,04	4,91	6,22	2,68	4,01	3,81	1,58	3,27	0,97

		RPKM													
#GeneID	TF	lung	lymph node	ovary	pancreas	placenta	prostate	salivary gland	skin	small intestine	spleen	stomach	testis	thyroid	urinary bladder
2625	GATA3	1,84	3,5	0,0619	0,0337	19,5	2,72	4,24	54,6	0,787	3,89	0,685	0,184	1,24	20
2551	GABPA	8,86	11,3	9,41	1,33	11,1	7,5	2,53	5,02	7,38	9,64	5,54	11	13,9	9,57
2002	Elk1	4,96	6,07	19,1	1,27	5,24	6,06	2,42	4,11	4,37	9,77	3,82	13,8	4,71	5,66
6929	E47	4,26	12,8	5,21	0,921	3,95	4,05	2,03	4,83	5,77	10,3	4,84	16,2	3,39	4,86

#BioProject
PRJNA66167
Mouse

		RPKM														
#GeneID	TF	CNS E11,5	CNS E14	CNS E18	Large Intestine adult	adrenal adult	bladder adult	cerebellum adult	colon adult	cortex adult	duodenum adult	frontal Lobe adult	genital fat pad adult	heart adult	kidney adult	limb E14,5
14462	GATA3	2,67	3,7	1,43	0,322	6,38	23,1	0,263	0,322	0,0102	0,449	0,0212	5,31	0,161	4,21	2,19
14390	Gabpa	12,4	8,68	7,35	3,43	9,78	8,93	4,99	4,29	4,03	6,3	5,13	3,9	5,85	5,45	10,9
13712	Elk1	6,17	10,4	10,5	1,88	16,3	4,16	9,99	4,17	10,3	2,15	9,58	5,78	4,39	3,48	9,25
21423	E47 (Tcf3)	50,8	31,9	13,3	18,4	40,3	8,75	6,31	23,7	4,29	26,8	5,8	13,9	5,08	8,84	49,2

		RPKM														
#GeneID	TF	liver E14	liver E14,5	liver E18	liver adult	lung adult	mammary gland adult	ovary adult	placenta adult	small intestine adult	spleen adult	stomach adult	subcutaneous fat pad adult	testis adult	thymus adult	whole brain E14,5
14462	GATA3	0,189	0,179	0,158	0,0962	2,91	22	0,39	3,5	0,484	2,06	1,01	0,347	0,591	17,3	4,41
14390	Gabpa	8	6,49	6,29	4,06	5,94	5,2	8,28	8,11	3,49	3,35	4,45	5	4,45	5,87	7,9
13712	Elk1	1,28	1,57	1,83	1,61	5,64	8,41	12,5	2,45	1,55	4,6	3,62	8,36	0,775	5,92	11,5
21423	E47 (Tcf3)	19,9	24,5	11,8	4,84	25,3	31,7	53,9	7,35	18,6	58,4	13,5	19,7	3,78	71,9	26,2

Supplementary Table S5 Reference numbers of the sequences used in this study

Scientific name	Common name	GABPA Refseq/uniprot accession number	TERT gene ID/Location on chromosome
Eschrichtius robustus	Gray whale	NA	NIPP01000104.1[573084..575084]
Tursiops truncatus	Common bottlenose dolphin	XP_004318161.1	101333195
Giraffa camelopardalis tippelskirchi	Masai giraffe	NA	LVKQ01181661.1[7541..9541]
Bos taurus	Cattle	Q0VC98	518884
Ovis aries	Sheep	AAC39262.1	100126866
Sus scrofa	Pig	XP_020927100.1	492280
Camelus dromedarius	Dromedary camel	XP_010975695.1	105102193
Ceratotherium simum simum	Southern White Rhinoceros	NA	101389836
Equus caballus	Horse	XP_014591809.1	100630695
Canis lupus familiaris	Dog	XP_013965264.1	403412
*Mustela putorius furo**	Domestic ferret (European polecat)	XP_004757497.1	NA
Mustela putorius eversmannii	Steppe Polecat	NA	101689866
Eptesicus fuscus	Big brown bat	XP_008143833.1	103305120
Myotis lucifugus	Small brown bat	XM_014461936.1	102424129
Mus musculus	House mouse	Q00422	21752
Rattus norvegicus	Brown rat	D4ACQ9	301965
*Peromyscus maniculatus bairdii**	Deer mouse	XP_015861914.1	NA
Peromyscus maniculatus sonoriensis	Sonoran Deer Mouse	NA	102928351
Castor canadensis	American beaver	JAV37339.1	109698261
Heterocephalus glaber	Naked mole-rat	XP_004842281.1	101706965
Cavia porcellus	Guinea pig	XP_013005230.1	100732437
Mesocricetus auratus	Golden hamster	XP_012971658.1	101832590
Ochotona princeps	American pika	XP_004588659.1	101516388
Microcebus murinus	Gray mouse lemur		105876452
Macaca mulatta	Rhesus macaque	F7E124	709865
Pongo abelii	Sumatran orangutan	XP_009232128.1	ABGA01240670.1[1..1261]
Homo sapiens	Human	Q06546	7015
Papio anubis	Olive baboon	XP_003895635.1	101024790
*Elephantulus edwardii**	Cape elephant shrew	XP_006896098.1	102873131
Elephantulus rufescens	Long-eared Elephant shrew	NA	NA
Loxodonta africana	African elephant	XP_003410373.1	100676312
Ornithorhynchus anatinus	Duck-billed platypus	XP_028938340.1	102060413
Oncorhynchus mykiss	Rainbow trout	XP_021453533.1	110487844

Supplementary Table S6 References for the ChIP-Seq data presented in this study

**Name**	**Sample Accession**	**ID**
GABPA-FLAG ChIP-Seq in Modified mouse ES cells	GSM1861943	301861943
HudsonAlpha_ChipSeq_H1-hESC_GABP_PCR1x	GSM803424	300803424
HudsonAlpha_ChipSeq_MCF-7_GABP_v042211.1	GSM1010864	301010864
HudsonAlpha_ChipSeq_SK-N-SH_GABP_v042211.1	GSM1010739	301010739
GABPA ChIP-Seq Rep 2 in HepG2 cells	GSM1861925	301861925

Supplementary Table S7 List of primers and oligonucleotides

Experiment	Primer name	Aim	fwd/rev	Sequence
**Reporter assay**	Ovb0162	hTERT promoter -500	fwd	CTCTTACGCGTGCTAGCCCG*TCTAGA*GCCAAAGGGTCGCCGCACGCACCTG
	Ovb0163	hTERT promoter-500	rev	GCTTACTTAGATCGCAGATC*TCTAGA*TCGCGGGGGTGGCCGGGGCCAGGG
	Ovb0164	mTERT promoter -500	fwd	CTCTTACGCGTGCTAGCCCG*GCTAGC*CCTCACTGTCTGTGTCAACCACAG
	Ovb0165	mTERT promoter-500	rev	GCTTACTTAGATCGCAGATC*GCTAGC*TTGTGCTCAAGGCCGGGATGGGCC
	Ovb0166	rTERT promoter -500	fwd	CTCTTACGCGTGCTAGCCCG*AAGCTT*TAATCATGAAGCCTGGTTGGGAAAAAC
	Ovb0167	rTERT promoter-500	rev	GCTTACTTAGATCGCAGATC*AAGCTT*TCGTGCTCTAGGTCGGGATGAACC
	Ovb0168	cTERT promoter -500	fwd	CTCTTACGCGTGCTAGCCCG*AGATCT*GCGGCAAGTCCCCCGCACCAGGGAC
	Ovb0169	cTERT promoter-500	rev	GCTTACTTAGATCGCAGATC*AGATCT*TGGCGTGGGAGGGTTCAGCCAGGC
	Ovb0170	pGL3	fwd	GATCTGCGATCTAAGTAAGCTTGGC
	Ovb0171	pGL3	rev	CGGGCTAGCACGCGTAAGAG
**Mutagenesis of promoter regions**	Ovb0172	mTERT promoter mutation	fwd	CCCTCCCGCTACAACGCTTGGTCCGCCTGAATC
	Ovb0173	mTERT promoter mutation	rev	GCGGGGTCGCAGCAATAGTACTTTTC
	Ovb0174	rTERT promoter mutation	fwd	CCCTCCCGCTACAACGCTTAATTCTCTTGGGTCCC
	Ovb0175	rTERT promoter mutation	rev	GCGGGGACGCAGCTGTGTGATGGTC
	Ovb0176	cTERT promoter mutation	fwd	CCCTTCCGGGCCCTTGCCCCGCCCCAACTTG
	Ovb0177	cTERT promoter mutation	rev	GCGGGGCTCGCGGAGGAAGGGGCG
	Ovb0196	hTERT promoter mutation (-146 to wild type)	fwd	CCCGGGTCCCCGGCCCAGCC
	Ovb0197	hTERT promoter mutation	rev	AGGGGTCGGGACGGGGCGGG
**Cloning of pET16-hisGABPA**	Ovb0108	pET16	fwd	CTCGAGGATCCGGCTGCTAACAAAG
	Ovb0139	pET16	rev	CATATGACGACCTTCGATATGGCCGCTGCTG
	Ovb0178	GABPA	fwd	GCGGCCATATCGAAGGTCGTCATATGACTAAAAGAGAAGCAGAGGAGCTG
	Ovb0179	GABPA	rev	CTTTGTTAGCAGCCGGATCCTCGAGAGATCTCAATTATCCTTTTCCGTTTGCAGAG
**Cloning of pET16-hisGFP-GABPA**	Ovb0198	GFP	fwd	GCGGCCATATCGAAGGTCGTCGTAAAGGAGAAGAACTTTTCAC
	Ovb0245	GFP	rev	CGATTGGAAGTAGAGGTTCTCGCTACCGCCTCCACCAACTGCTGCAGCGTAGTTTTCG
	Ovb0202	GABPA-pET16	fwd	GAGAACCTCTACTTCCAATCGACTAAAAGAGAAGCAGAGGAGCTG
	Ovb0201	GABPA-pET16	rev	ACGACCTTCGATATGGCCGCTG
**ChIP primers**	Ovb0146	mTERT promoter	fwd chip	AGTACTATTGCTGCGACCCC
	Ovb0147	mTERT promoter	rev chip	GGGAACCAAGATGCAAGGGT
	Ovb0152	mCOXVb promoter	fwd chip	GCGTCTACTTTTAGCGCTGC
	Ovb0153	mCOXVb promoter	rev chip	CCACTCCGCGAAGTAACCTT
	Ovb0158	m-intergen	fwd chip	GTTACAGAGCTCCAGCCCAC
	Ovb0159	m-intergen	rev chip	CAGGTATCGTCTCACGGCAG

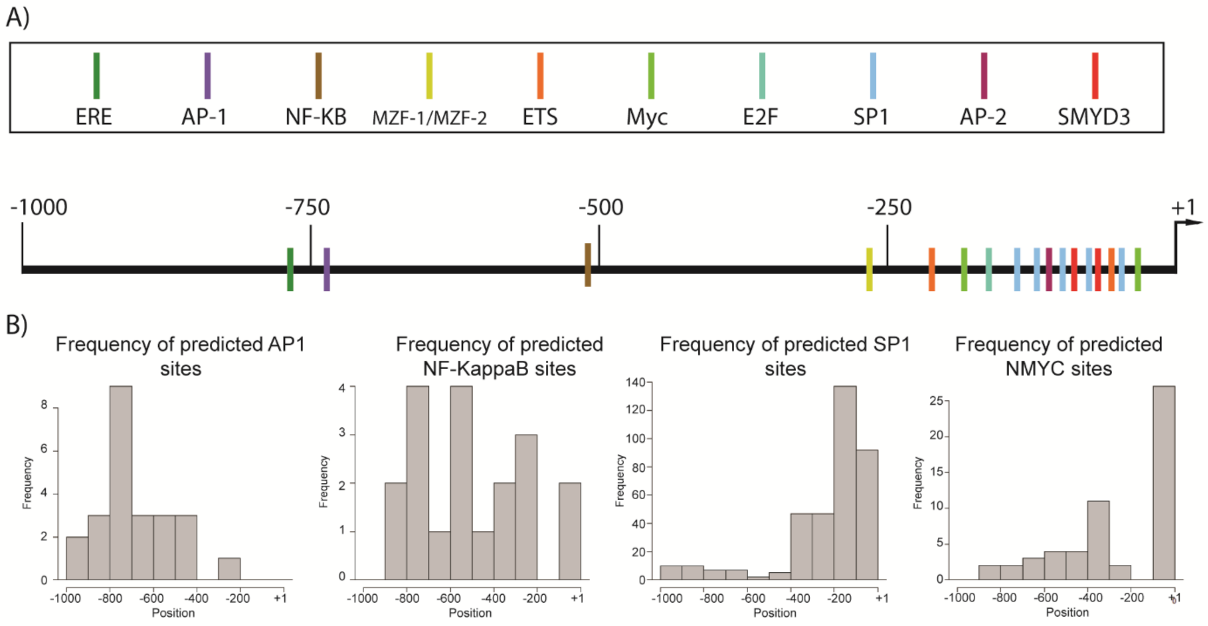

Supplementary Fig S4 Comparison of known and predicted transcription factor binding sites. (A) Transcription factor binding sites on human TERT promoter based on the literature [2–4]. (B) Histograms of some predicted transcription factors.

Supplementary Table S8 Scores of transcription binding site prediction using different prediction methods

Matrice	Species	Position	TFbind score	Match Score	FIMO p-value
V$NRF2_01	Elephantulus_edwardii	32		0,953	
V$NRF2_01	Microcebus_murinus	76	0,920058		4,41E-05
V$NRF2_01	Eschrichtius_robustus	89	0,920058		4,41E-05
V$NRF2_01	Camelus_dromedarius	112	0,920058		4,41E-05
V$NRF2_01	Heterocephalus_glaber	153		0,951	
V$NRF2_01	Elephantulus_edwardii	252	0,903585		7,70E-05
V$NRF2_01	Ceratotherium_simum_simum	296	0,920058		4,41E-05
V$NRF2_01	Giraffa_camelopardalis_tippelskirchi	357	0,924419		3,85E-05
V$NRF2_01	Cavia_porcellus	396	0,936047	0,946	2,37E-05
V$NRF2_01	Heterocephalus_glaber	407	0,936047	0,946	2,37E-05
V$NRF2_01	Sus_scrofa	682	0,952519	0,96	1,04E-05
V$NRF2_01	Microcebus_murinus	698	0,920058	0,945	3,27E-05
V$NRF2_01	Rattus_norvegicus	742	0,914729		4,53E-05
V$NRF2_01	Rattus_norvegicus	834	0,936531	0,959	2,24E-05
V$NRF2_01	Cavia_porcellus	834	0,920058	0,945	3,27E-05
V$NRF2_01	Mus_musculus	835	0,936531	0,959	2,24E-05
V$NRF2_01	Peromyscus_maniculatus_bairdii	836	0,936531	0,959	2,24E-05
V$NRF2_01	Mesocricetus_auratus	847	0,962209	0,962	8,31E-06
V$NRF2_01	Heterocephalus_glaber	850	0,920058	0,945	3,27E-05
V$GATA3_01	Myotis_lucifugus	56	0,931325		0,000249
V$GATA3_01	Mus_musculus	143	0,937528		0,000119
V$GATA3_01	Mus_musculus	174	0,920691		0,000546
V$GATA3_01	Giraffa_camelopardalis_tippelskirchi	275	0,905627		0,000476
V$GATA3_01	Giraffa_camelopardalis_tippelskirchi	314	0,935755		0,000199
V$GATA3_01	Mustela_putorius	340	0,929109		0,000187
V$GATA3_01	Rattus_norvegicus	449	0,920691		0,000546
V$GATA3_01	Rattus_norvegicus	627	0,920691		0,00056
V$GATA3_01	Elephantulus_edwardii	646	0,90607		0,000486
V$GATA3_01	Camelus_dromedarius	656	0,907399		0,00085
V$GATA3_01	Loxodonta_africana	751	0,954807		2,67E-05
V$GATA3_01	Eptesicus_fuscus	803	0,902525		0,000581
V$GATA3_01	Loxodonta_africana	837	0,931768		9,66E-05
V$GATA3_01	Sus_scrofa	850	0,916704		0,00014
V$GATA3_01	Elephantulus_edwardii	861	0,946389		4,35E-05
V$GATA3_01	Mus_musculus	890	0,900753		0,000836
V$Elk1_02	Elephantulus_edwardii	31		0,972	0,000382
V$Elk1_02	Heterocephalus_glaber	152	0,905724	0,991	2,52E-05
V$Elk1_02	Bos_taurus	277		0,952	0,000151
V$Elk1_02	Bos_taurus	297		0,941	
V$Elk1_02	Giraffa_camelopardalis_tippelskirchi	354		0,942	
V$Elk1_02	Cavia_porcellus	395	0,947964	0,993	1,81E-06
V$Elk1_02	Heterocephalus_glaber	406	0,941537	0,993	4,25E-06

V$Elk1_02	Sus_scrofa	681	0,925314	0,992	4,56E-06
V$Elk1_02	Elephantulus_edwardii	696		0,959	0,000486
V$Elk1_02	Microcebus_murinus	697	0,900826		2,30E-05
V$Elk1_02	Ochotona_princeps	728	0,941537		2,08E-06
V$Elk1_02	Rattus_norvegicus	741		0,957	0,000382
V$Elk1_02	Loxodonta_africana	791		0,959	0,000105
V$Elk1_02	Cavia_porcellus	831		0,974	9,39E-05
V$Elk1_02	Rattus_norvegicus	833		0,974	0,000359
V$Elk1_02	Mus_musculus	834		0,947	0,000359
V$Elk1_02	Peromyscus_maniculatus_bairdii	835		0,945	0,000561
V$Elk1_02	Mesocricetus_auratus	837		0,944	0,000853
V$Elk1_02	Heterocephalus_glaber	847		0,974	0,00014
V$Elk1_02	Peromyscus_maniculatus_bairdii	895		0,945	0,000287
V$E47_01	Equus_caballus	26		0,949	7,77E-05
V$E47_01	Ovis_areis	125		0,946	0,000158
V$E47_01	Macaca_mulatta	127	0,92045		1,11E-05
V$E47_01	Papio_anubis	127	0,92045		1,11E-05
V$E47_01	Bos_taurus	184	0,948775	0,967	1,65E-06
V$E47_01	Eschrichtius_robustus	213	0,948775	0,967	1,65E-06
V$E47_01	Tursiops_truncatus	215	0,948775	0,967	1,65E-06
V$E47_01	Castor_canadensis	222		0,971	4,89E-05
V$E47_01	Mustela_putorius	229		0,949	9,30E-05
V$E47_01	Giraffa_camelopardalis_tippelskirchi	245	0,919446		9,62E-06
V$E47_01	Canis_lupus	267	0,918039		1,00E-05
V$E47_01	Peromyscus_maniculatus_bairdii	597	0,955404	0,984	2,36E-06
V$E47_01	Mesocricetus_auratus	641	0,912616		3,11E-05
V$E47_01	Ceratotherium_simum_simum	667	0,919646		7,78E-06
V$E47_01	Canis_lupus	734		0,942	3,27E-05
V$E47_01	Equus_caballus	749	0,975291	0,994	9,86E-08
V$E47_01	Mustela_putorius	799		0,942	1,83E-05

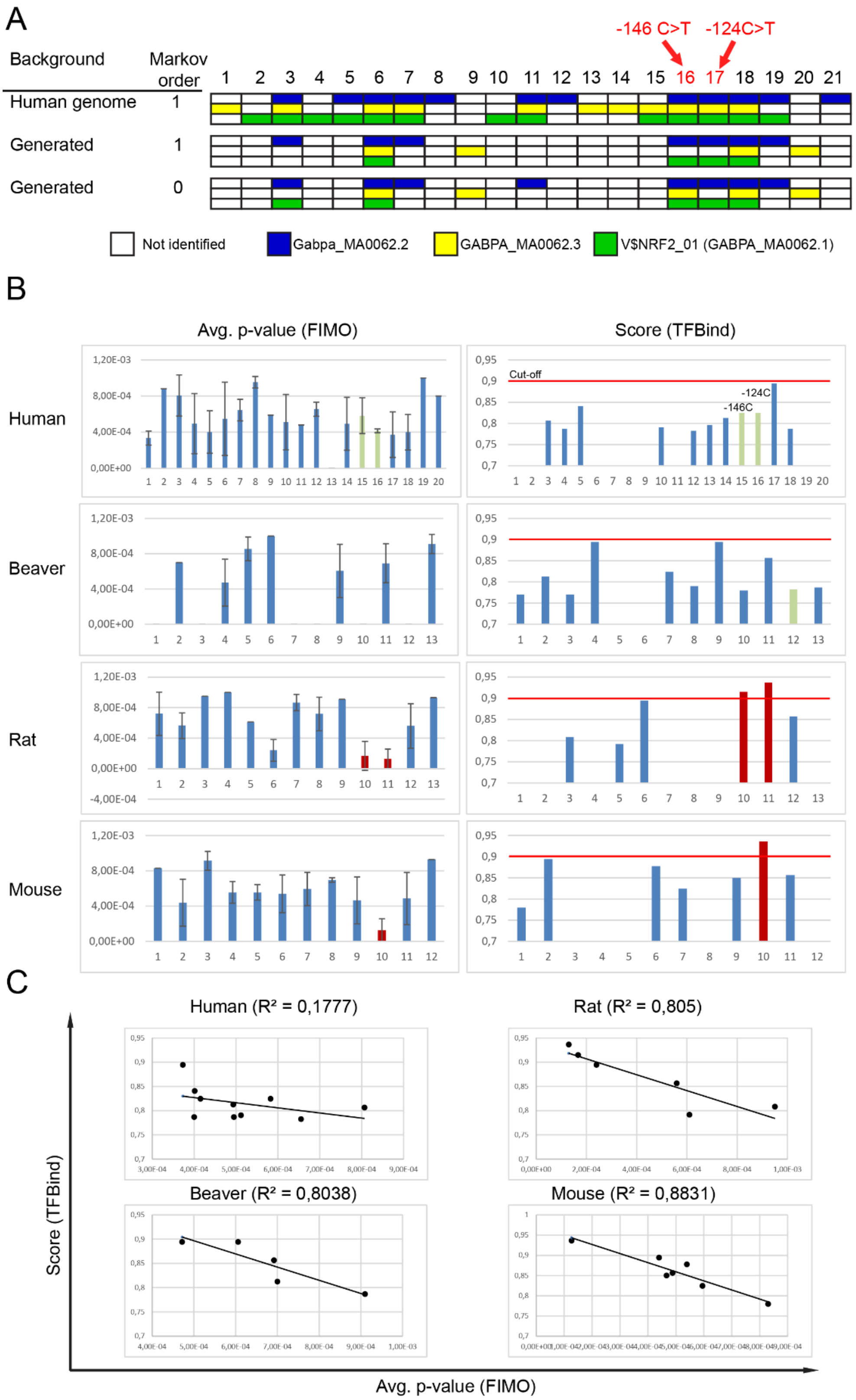

Supplementary Fig S5 GABPA FIMO results comparisons. (A) GABPA binding sites identified on double mutant TERT promoter by FIMO using human genome and "generated" background to filter false positives. The generated background is calculated based on the promoter sequences provided. The

three available GABPA matrices are marked accordingly. The identification of the two confirmed binding sites (-124 C>T and -146C>T) depends on the selected background in the case of GABPA_MA0062.3 matrix, which represents chip-seq data from *Homo sapiens*. (B) Due to the experienced differences we ran FIMO with various settings (human and generated background, both with Markov order 0-2) and calculated an average for the p-values of each identified sites. The low p-values were associated with high TFbind score (red). The green colours represent weak binding sites which could be modified to strong binding sites (Supplementary 1). (C) The average p-values from FIMO correlate with the TFbind scores in most cases.

Supplementary Note S1 Codes

#R code for bootstrapping

```
#set working directory!

#load header - names of the TFBS and telomer state

fejlec=read.table('fejlec-bootstraphoz.txt', header=F)

#load data (the firs line is the names of the transcription factors along wit telomere state (telstate). The following lines are the frequency of TFBS in each species)

adat=read.table('bootstraphoz.txt', header=T)

attach(adat)

#number of iteration

x=5000

testcounter=NULL

#only the 60 most abundant sites are studied

for (i in 1:60) {

	#separating the data based on telomerase negative (A) or telomerase positive (B)

	A=adat[telstate==0, i]

	B=adat[telstate==1, i]

	test=0

	for (i in 1:x){

		#random sampling, calculation of median

		a=median(sample(A, size=length(A), replace=TRUE))

		b=median(sample(B, size=length(B), replace=TRUE))

		#hypothesis testing (">" or "<" or "==") in this case "==" run all 3 separately

		if (a==b) {

			test=test+1

		}

	}

	# saving of p-value

	testcounter= c(testcounter, test/x)

}

# exporting data

names=NULL

for (i in 1:60){
```

```
            C=toString(fejlec[1,i])
            nevek=c(names, C)
            }
output = rbind(names, testcounter)
write.table(output, "c:/R-working/mydata.txt", sep="\t")
```

# Python code of k-means and PCA analysis

```
# importing the Python libriaries for the analysis
import pandas as pd
from sklearn.cluster import KMeans
import matplotlib.pyplot as plt
from sklearn.decomposition import PCA
import numpy as np
#creating an empty dataframe
datf=pd.DataFrame()
#reading data file to pandas dataframe - the file path is an example
df = pd.read_csv(r"d:\file_path\data.txt",sep="\t", engine='python')
# setting the number of iterations
q=0
w=q+20
# creating a secondary variable for the original dataframe
df2 = df
# iteration for multiple runs
for j in range(q,w):
# potential iterations in the number of clusters (k) we considered to choose k=5,
# in case of testing multiple values of k, only the last iteration will be plotted,
# however the cluster labels would be available for previous iterations as well
    for k in range (5,6):
# k-means clustering
        kmen=KMeans(n_clusters=k)
        kmen.fit(df2.drop(["animal_name"],axis=1))
        df["kmeansn"+str(k)]=kmen.labels_
```

```
    datf["kmeansn"+str(j)+str(k)]=kmen.labels_
# "blanking" PCA - variables between iterations
    pca_=""
    Pca_2d=""
# Principal Component analysis
    pca_ = PCA(n_components=2).fit(df2.drop(['animal_name'],axis=1))
    pca_2d = pca_.transform(df2.drop(['animal_name',],axis=1))
# creating a scatter plot based on k-means and PCA analyses
    plt.figure
    plt.scatter(pca_2d[:,0],pca_2d[:,1],s=160, c=df["kmeansn"+str(k)])
    plt.xlabel('PCA-1 ('+str(int(np.rint(pca_.explained_variance_ratio_[0]*10000))/100)+"%)")
    plt.ylabel('PCA-2 ('+str(int(np.rint(pca_.explained_variance_ratio_[1]*10000))/100)+"%)")
# annotate the dots on scatterplot
    z=pca_2d[:,0]
    y=pca_2d[:,1]
    n=df.animal_name
    for i, txt in enumerate(n):
        plt.annotate(txt, (z[i], y[i]))
# viewing the plot
    plt.show()
# saving the data and labels
    df.to_csv(r"d:\file_path\file_name"+str(j)+"_"+str(k)+".txt", sep="\t")
# saving the figures in high quality pdf for publication
    plt.savefig(r"d:\file_path\file_name"+str(j)+"_"+str(k)+".pdf",dpi=300)
# saving the figures in png format for quick review
    plt.savefig(r"d:\file_path\file_name"+str(j)+"_"+str(k)+".png")
# saving the cluster labels in a single file
datf.to_csv(r"d:\file_path\summary.txt", sep="\t")
```

References

1. Suzuki, F. *et al.* Functional interactions of transcription factor human GA-binding protein subunits. *J. Biol. Chem.* (1998). doi:10.1074/jbc.273.45.29302

2. Lewis, K. A. & Tollefsbol, T. O. Regulation of the telomerase reverse transcriptase subunit through epigenetic mechanisms. *Frontiers in Genetics* (2016). doi:10.3389/fgene.2016.00083

3. Liu, T., Yuan, X. & Xu, D. Cancer-specific telomerase reverse transcriptase (Tert) promoter mutations: Biological and clinical implications. *Genes (Basel).* **7**, 1–18 (2016).

4. Khattar, E. & Tergaonkar, V. Transcriptional regulation of telomerase reverse transcriptase (TERT) by MYC. *Frontiers in Cell and Developmental Biology* (2017). doi:10.3389/fcell.2017.00001
